# A high fat diet with a high C18:0/C16:0 ratio induced worse metabolic and transcriptomic profiles in C57BL/6 mice

**DOI:** 10.1186/s12944-020-01346-z

**Published:** 2020-07-21

**Authors:** Liqiang Wang, Fei Xu, Zhenfeng Song, Dan Han, Jingyi Zhang, Linjun Chen, Lixin Na

**Affiliations:** 1 Food Hygiene and Nutrition Department, Shanghai University of Medicine & Health Science, Shanghai, 201318 P. R. China; 2 Collaborative Innovation Center for Biomedicine, Shanghai University of Medicine & Health Science, Shanghai, 201318 P. R. China; 3 Department of Nutrition and Food Hygiene, Public Health College, Harbin Medical University, Harbin, 150081 P. R. China

**Keywords:** High fat diet, Palmitic acid, Stearic acid, Metabolic profile, Transcriptomic profile, Long non-coding RNA, microRNA, mRNA

## Abstract

**Background:**

Differential effects of individual saturated fatty acids (SFAs), particularly stearic acid (C18:0), relative to the shorter-chain SFAs have drawn interest for more accurate nutritional guidelines. However, specific biologic and pathologic functions that can be assigned to particular SFAs are very limited. The present study was designed to compare changes in metabolic and transcriptomic profiles in mice caused by a high C18:0 diet and high palmitic acid (C16:0) diet.

**Methods:**

Male C57BL/6 mice were assigned to a normal fat diet (NFD), a high fat diet with high C18:0/C16:0 ratio (HSF) or an isocaloric high fat diet with a low C18:0/C16:0 ratio (LSF) for 10 weeks. An oral glucose tolerance test, 72-h energy expenditure measurement and CT scan of body fat were done before sacrifice. Fasting glucose and lipids were determined by an autobiochemical analyzer. Blood insulin, tumor necrosis factor-α (TNF-α), and interleukin-6 (IL-6) levels were measured by enzyme-linked immunosorbent assay methods. Free fatty acids (FFAs) profiles in blood and liver were determined by using gas chromatography-mass spectrometry. Microarray analysis was applied to investigate changes in transcriptomic profiles in the liver. Pathway analysis and gene ontology analysis were applied to describe the roles of differentially expressed mRNAs.

**Results:**

Compared with the NFD group, body weight, body fat ratio, fasting blood glucose, insulin, homeostasis model assessment of insulin resistance (HOMA-IR), triglyceride, IL-6, serum and liver FFAs including total FFAs, C16:0 and C18:0 were increased in both high fat diet groups and were much higher in the HSF group than those in the LSF group. Both HSF and LSF mice exhibited distinguishable long non-coding RNA (lncRNA), microRNA and mRNA expression profiles when compared with those of NFD mice. Additionally, more differentially expressed lncRNAs and mRNAs were observed in the HSF group than in the LSF group. Some biological functions and pathways, other than energy metabolism regulation, were identified as differentially expressed mRNAs between the HSF group and the LSF group.

**Conclusion:**

The high fat diet with a high C18:0/C16:0 ratio induced more severe glucose and lipid metabolic disorders and inflammation and affected expression of more lncRNAs and mRNAs than an isocaloric low C18:0/C16:0 ratio diet in mice. These results provide new insights into the differences in biological functions and related mechanisms, other than glucose and lipid metabolism, between C16:0 and C18:0.

## Introduction

Excessive intake of a high fat diet commonly leads to obesity, abnormal blood lipid profiles and even insulin resistance [1, 2]. Increased plasma saturated fatty acids (SFAs), induced by a high fat diet, are mainly responsible for these pathologic progresses [3–5]. Therefore, control of dietary SFAs intake has been widely applied to prevent metabolic risks and cardiovascular diseases [6, 7].

However, some studies have suggested that dietary fatty acid composition rather than dietary fat content contributes more to insulin sensitivity and health. For example, palmitic acid (C16:0) and C18:0 are the most common and abundant long chain SFAs in food and the human body, and C16:0 can be converted to C18:0 in the body [8]. A high fat diet with an increase in C18:0 has been found to induce a metabolic state favoring lower oxidative metabolism and severe hepatic insulin resistance in mice compared with an isocaloric high fat diet [9]. In mice deficient for Elovl6, a gene encoding the elongase that catalyzes the conversion of C16:0 to C18:0, the level of C18:0 decreased while the level of C16:0 increased in serum and liver, and the mice became obese and developed hepatosteatosis but were protected from insulin resistance when fed a high fat diet [10]. Therefore, differential effects of individual SFAs, particularly stearic acid (18:0), relative to the shorter-chain SFAs have drawn much interest for accurate nutritional guidelines [11]. However, specific biologic and pathologic functions that can be assigned to particular SFAs are very limited, and further research needs to be carried out [12].

According to the current evidence, there are likely two reasons for the different effects on insulin resistance of C18:0 and C16:0. On one hand, C18:0, compared to C16:0, is poorly incorporated into triacylglycerol in the liver [13]. On the other hand, the efficiency of SFA oxidation has been demonstrated to be related to its chain length: the longer the chain length of the SFAs is, the slower the rate of oxidation would become [14, 15]. Thus, when C18:0 increases in the body, intermediate lipid metabolites, such as diacylglycerols and ceramides, are more likely to accumulate and have been reported to interfere with the insulin signaling pathway [16–19].

As the structure and metabolism between C16:0 and C18:0 are not exactly the same, they may play many different roles in the body, except for some similar functions in energy metabolism [20]. In the present study, the mice were fed isocaloric high fat diets with different C18:0/C16:0 ratios to induce obesity and insulin resistance. Metabolic parameters in serum and liver were determined, and microarray analysis of the liver was done to investigate the changes in long non-coding RNA (lncRNA), microRNA and mRNA expression. The aim of this study was: (1) to systematically compare the different effects between C16:0 and C18:0 at the metabolic and even gene transcriptional levels in mice in order to provide more details regarding their pathophysiological activities, and (2) to provide supporting evidence to control the dietary C18:0/C16:0 ratio for accurate nutrition and health.

## Materials and methods

### Animals

Eight-week-old male C57BL/6 mice (18–22 g) were purchased from Beijing Vital River Laboratory Animal Technology Co., Ltd. (Beijing, China). The mice were housed individually in pathogen-free metabolic cages in an environmentally controlled room at 21 ± 2 °C and 50 ± 5% humidity with 12-h light/dark cycles; lights were on at 0700 h and off at 1900 h. The study was approved by the Institutional Animal Care Committee from Harbin Medical University and conducted in accordance with the University guidelines for the care and use of laboratory animals (approval number:12–0003).

### Treatments

The mice had ad libitum access to water and standard laboratory chow (Beijing Keao Xieli Feed CO., LTD., Beijing, China). After acclimation for 1 week, the mice were randomly divided into 3 groups (*n* = 20 for each group). The mice in the normal fat diet group (NFD) were fed a normal standard laboratory chow diet (15% of the energy in the form of fat). The mice in the high C18:0/C16:0 group (HSF) were fed a high fat diet (36% of the energy in the form of fat) with lard as the major source of C18:0. The mice in the low C18:0/C16:0 group (LSF) were fed a high fat diet (36% of the energy in the form of fat) with palm oil as the major source of C16:0 (Table S1). The C18:0/C16:0 ratio in high fat diets was determined by a gas chromatography-mass spectrometry. The C18:0/C16:0 ratio was 1:2 in HSF diet and 1:8 in LSF diet. Food intake and body weight were measured daily and weekly, respectively. At the end of the 10th week, blood was collected directly from the heart by cardiac puncture under deep anesthesia. Livers and pancreas were weighed and stored at − 80 °C for further use.

### Energy expenditure measurement

Energy expenditure was assessed using the TSE PhenMaster system (TSE Systems GmbH, Bad Homburg, Germany). At the end of the 9th week, six mice were randomly picked from each group and placed in the metabolic chambers and acclimated for 24 h. Energy expenditure analysis was performed for consecutive 72 h. Oxygen consumption (VO_2_) and carbon dioxide production (VCO_2_) were measured at 27-min intervals. VO_2_ and VCO_2_ values were in mL/h. The total energy expenditure (EE) was calculated from the sum of fat and carbohydrate oxidation. Respiratory exchange rate (RER) was calculated as VCO_2_/VO_2_. Fat and carbohydrate oxidation rates were calculated according to the following equation [9]:

$$ \mathrm{Carbohydrate}\ \mathrm{oxidation}\ \mathrm{rate}\ \left(\mathrm{kcal}/\mathrm{h}\right)=\left(\left(4.585\times {\mathrm{VCO}}_2\right)\hbox{-} \left(3.226\times {\mathrm{VO}}_2\right)\right)\times 4/1000 $$

$$ \mathrm{Fat}\ \mathrm{oxidation}\ \mathrm{rate}\ \left(\mathrm{kcal}/\mathrm{h}\right)=\left(\left(1.695\times {\mathrm{VO}}_2\right)\hbox{-} \left(1.701\times {\mathrm{VCO}}_2\right)\right)\times 9/1000 $$

### Body fat distribution analysis

At the end of the 10th week, six mice were randomly picked from each group and were scanned with a Latheta LCT-200 (Hitachi-Aloka Medical, Tokyo, Japan) in a prone position to image the fat distribution. Visceral fat, subcutaneous fat, and muscle mass were calculated based on the scanned CT value. Total fat ratio = total fat mass/(total fat mass + muscle mass) × 100%. Liver fat ratio = (muscle CT value–liver CT value)/(muscle CT value–fat CT value) × 100%.

### Oral glucose tolerance test (OGTT) and homeostasis model assessment of insulin resistance (HOMA-IR) index

At the end of the 10th week, OGTT was done in each group before sacrifice. Following a 15-h fasting, 25% glucose (w/v) was given by gavage at a dose of 0.2 mL/10 g·BW. Blood sample was collected from the tail vein at 0, 30, 60, 90 and 120 min. Insulin resistance was estimated by HOMA-IR based on fasting glucose and insulin levels as follows: HOMA-IR = fasting glucose (mmol/L) × fasting insulin (mU/L)/ 22.5.

### Measurement of blood biochemical parameters and inflammatory markers

Fasting blood glucose, total cholesterol (TC), triglyceride (TG), high density lipoprotein cholesterol (HDL-c) and low density lipoprotein cholesterol (LDL-c) were determined with a Hitachi 7100 auto-biochemical analyzer (Hitachi-Aloka Medical, Tokyo, Japan). Blood insulin (R&D Systems, Abnova, USA), tumor necrosis factor-α (TNF-α) (Sigma-Aldrich.com, St. Louis, MO, USA), and interleukin-6 (IL-6) (Sigma-Aldrich.com, St. Louis, MO, USA) were measured by enzyme linked immunosorbent assay methods.

### Measurement of fatty acids profile in serum and liver

The levels of fatty acids in serum and liver of mice were detected by a Gas Chromatography-Mass Spectrometry (TRACE GC/PolarisQ MS, Thermo Finnigan, San Jose, USA) as previously described [21] (Suppl. Methods- GC-MS conditions and method performance).

### Detection of cell ultra-structural changes in liver and pancreas

Liver and pancreas tissues were fixed by 2.5% glutaraldehyde solution and 1% osmium acid solution. After dehydration using ethanol and acetone, tissues were embedded in acetone and embedding solvent. Ultra-thin sections were prepared by a Reichert-Jung Ultracut E ultramicrotome (Reichert, Vienna, Austria) and stained with uranyl acetate and lead citrate solution. Transmission electron microscopy was employed to observe and analyze the changes of mitochondria in liver and insulin granules in pancreas.

### Analysis of lncRNA/microRNA/mRNA expression in liver

Gene expression in liver of mice (*n* = 3 per group) was determined by Kangcheng Bio-tech Inc. (Shanghai, China). Total RNA from each sample was quantified using the NanoDrop ND-1000 and the RNA integrity was assessed using standard denaturing agarose gel electrophoresis. For microarray analysis, Agilent Array platform was employed. The sample preparation and microarray hybridization were performed based on the manufacturer’s standard protocols with minor modifications (Suppl. Methods- Microarrays methods). For lncRNAs and mRNAs, Agilent Feature Extraction software (version 11.0.1.1) was used to analyze the acquired array images. Quantile normalization and subsequent data processing were performed using the GeneSpring GX v11.5.1 software package (Agilent Technologies). For miRNAs, scanned images were then imported into GenePix Pro 6.0 software (Axon Instruments, Union City, CA, USA) for grid alignment and data extraction. Differentially expressed genes with statistical significance were identified through Volcano Plot filtering. For lncRNAs, those with fold changes ≥2.0 and *P*-value < 0.05 were considered significantly different. For microRNAs and mRNAs, those with fold changes ≥1.5 and *P*-value < 0.05 were considered significantly different. Hierarchical clustering was also performed to show the distinguishable lncRNAs, microRNAs and mRNAs expression pattern among samples. Pathway analysis and gene ontology (GO) analysis were applied to describe the roles of these differentially expressed mRNAs played in these biological pathways or GO terms. All the microarray data have been deposited in a public and community-supported repository, prior to publication of this associated manuscript (https://www.ebi.ac.uk/fg/annotare/). These data (differentially expressed lncRNAs in liver among NFD, LSF and HSF groups (E-MTAB-9167), differentially expressed microRNAs in liver among NFD, LSF and HSF groups (E-MTAB-9168), differentially expressed mRNAs in liver among NFD, LSF and HSF groups (E-MTAB-9169)) are to be made public for free at the Arrayexpress platform after the present manuscript is published.

### Statistical analysis

All data were expressed as the mean ± SEM. SPSS v21.0 (Beijing Stats Data Co. Ltd., Beijing China) was used for statistical analysis. Comparisons among 3 groups in body weight and fat distribution, energy metabolism, blood biochemical parameters, inflammatory factors, and FFAs levels were performed using ANOVA with a Bonferroni post hoc test for difference between 2 groups (HSF vs. NFD, LSF vs. NFD, HSF vs. LSF, respectively). *P* < 0.05 was considered to be statistically significant.

## Results

### The effect of dietary C18:0/C16:0 ratios on body weight and body fat content

**Fig. 1:**
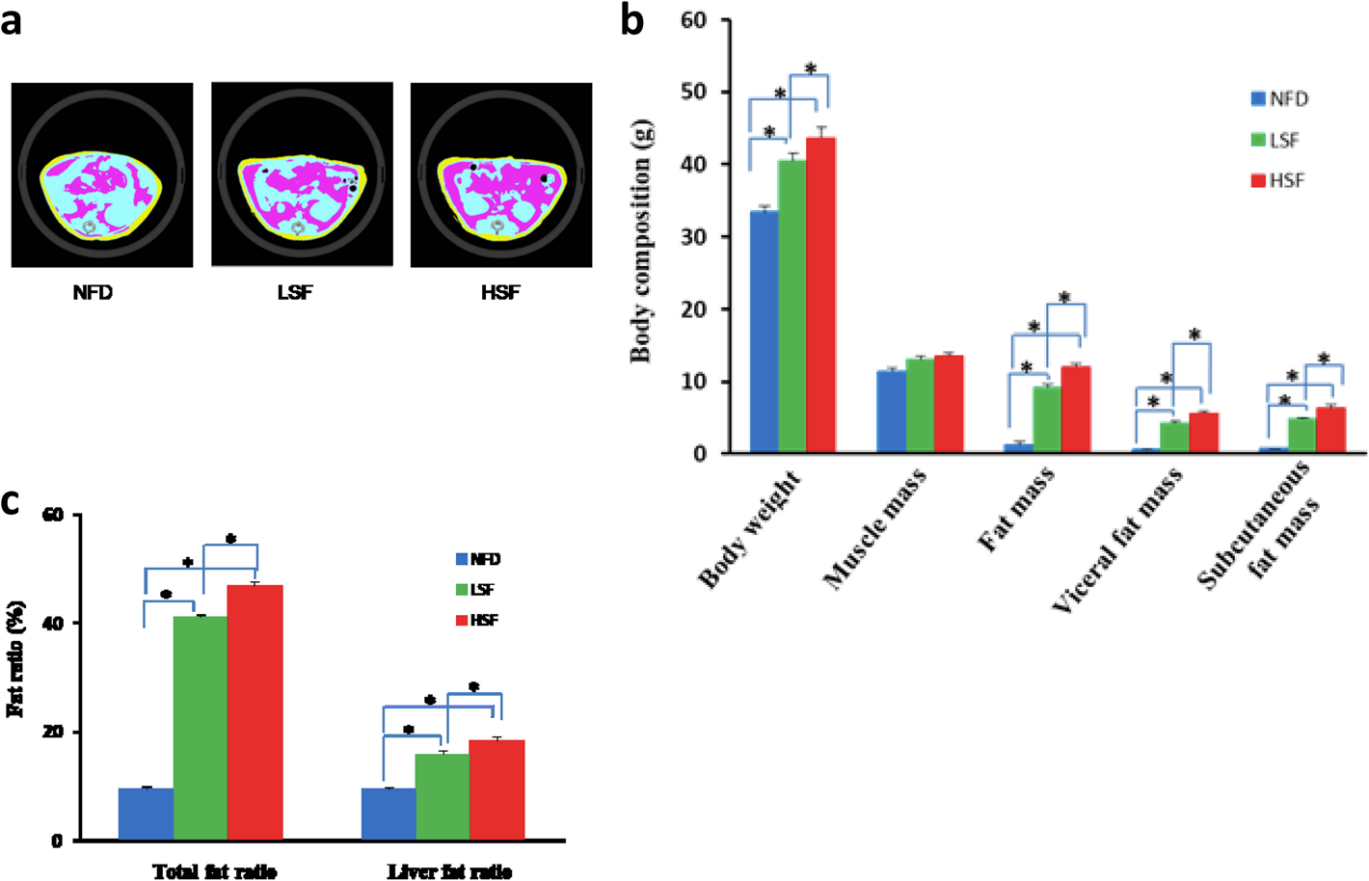
Body fat content of mice. **a**, CT images of mice fat distribution in the 3 groups. **b**, Comparisons of mice body composition among groups (*n* = 6 for each group). **c**, Comparisons of fat radios among groups (*n* = 6 for each group). NFD, normal fat diet group; HSF, high stearic acid diet group (C18:0/ C16:0 = 1:2); LSF, low stearic acid diet group (C18:0/ C16:0 = 1:8). ^*^P < 0.05, compared between two groups. Yellow for subcutaneous fat and pink for visceral fat in Fig. 1a

At the end of the 6th week, the body weight of mice in the HSF group was higher than that of mice in the LSF group (Table S2). No significant changes in diet intake were observed among the three groups (data not shown). CT images of body fat distribution showed the richest visceral and subcutaneous fat existed in the HSF group, followed by the LSF group, which were much higher than those in the NFD group (Fig. 1a). The total body fat ratio and liver fat ratio were also higher in the HSF group than those in the LSF group (Fig. 1b, c, Table S3 and S4).

### The effect of dietary C18:0/C16:0 ratios on energy metabolism

There was no difference in energy expenditure among the 3 groups (Fig. S1a). Carbohydrate oxidation in the HSF and LSF groups was significantly lower while fat oxidation was much higher than that in the NFD group (Fig. S1b, c). Moreover, fat oxidation in the LSF group was significantly higher than that in the HSF group, indicating a lower oxidation rate of C18:0 than C16:0. There was a decreasing trend of RER in the HSF and LSF groups compared with that in the NFD group (Fig. S1d).

### The effect of dietary C18:0/C16:0 ratios on blood biochemical parameters, glucose tolerance and inflammatory markers

Mice in both high fat diet groups exhibited decreased glucose tolerance, but the glucose tolerance was impaired more severely in the HSF group than that in the LSF group (Fig. S2). Fasting blood glucose, insulin, TC, TG, HDL-c, LDL-c, TNF-α, IL-6 and HOMA-IR in the high fat diet groups were significantly higher than those in the NFD group, and glucose, insulin, HOMA-IR, TG and IL-6 levels in the HSF group were higher than those in the LSF group (Table S5).

### The effect of dietary C18:0/C16:0 ratios on fatty acid profiles in serum and liver

The levels of serum total fatty acids, saturated fatty acids, unsaturated fatty acids, C16:0, C18:0, C18:1, and C18:3 were significantly higher in mice fed a high fat diet, and these fatty acids were much higher in the HSF group than those in the LSF group (Table 1). The changes in liver fatty acid profiles were similar to those in serum among the groups (Table S6).

**Table 1:** Serum fatty acids profile in mice

FAs (μg/mL)	NFD	LSF	HSF
C14:0	5.15 ± 0.97	5.55 ± 0.73	8.3 ± 2.13
C16:0	257.4 ± 78.21	330.05 ± 58.69^*^	377.67 ± 44.33^*#^
C16:1	37.07 ± 5.08	39.68 ± 8.51	52.18 ± 10.33^*^
C18:0	55.39 ± 42.35	80.86 ± 29.84^*^	120.08 ± 26.37^*#^
C18:1	159.06 ± 15.99	236.41 ± 22.68^*^	272.41 ± 23.83^*#^
C18:2	313.71 ± 54.58	256.11 ± 17.52^*^	286.88 ± 31.07
γ- C18:3	13.04 ± 8.41	8.42 ± 4.16	12.06 ± 5.64
C18:3	201.41 ± 43.73	82.31 ± 15.27^*^	114.14 ± 8.37^*#^
C20:2	35.96 ± 40.21	150.16 ± 98.72^*^	98.11 ± 46.96^*^
C20:4	81.72 ± 10.27	144.23 ± 37.63^*^	148.17 ± 49.17^*^
C20:5	6.02 ± 1.78	2.6 ± 1.04	6.66 ± 2.36
saturated fatty acid	317.94 ± 89.10	406.24 ± 98.47^*^	506.05 ± 69.91^*#^
unsaturated fatty acid	847.98 ± 145.63	885.06 ± 165.88	990.63 ± 130.54^*#^
total free fatty acids	1128.85 ± 243.39	1296.7 ± 129.58^*^	1444.79 ± 184.27^*#^

All values are presented as mean ± SEM (*n* = 10). NFD, normal fat diet group; HSF, high stearic acid diet group (C18:0/ C16:0 = 1:2); LSF, low stearic acid diet group (C18:0/ C16:0 = 1:8). ^*^ Compared with the NFD group, *P* < 0.05. ^#^ Compared with LSF group, *P* < 0.05

### The effect of dietary C18:0/C16:0 ratios on cell ultrastructure in liver and pancreas

Mitochondria in the high fat diet groups were severely distended compared with those in the NFD group, and mitochondrial distension in the HSF group was more serious than that in the LSF group. Some mitochondria in the HSF group showed an exvaginated inner mitochondrial membrane (Fig. S3a, b, c). For the pancreas, the amount of insulin granules decreased significantly in both the LSF and HSF groups, and there were obvious vacuoles induced by denatured insulin granules in the HSF group (Fig. S3d, e, f).

### Differentially expressed lncRNAs in the liver among groups

**Fig. 2:**
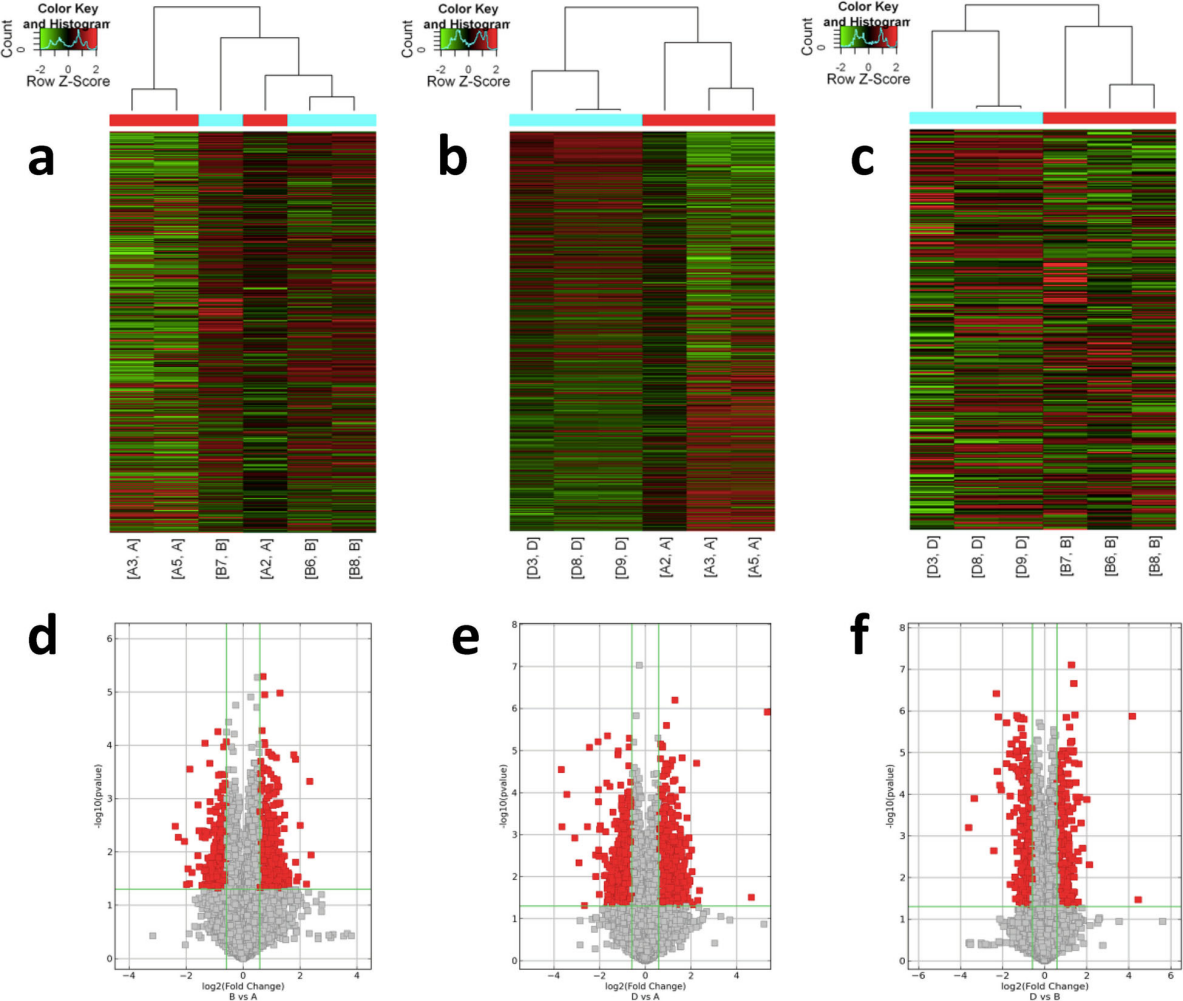
Dfferentially expressed lncRNAs in liver of mice. **a**-**c**, Differentially expressed lncRNAs in liver analyzed using hierarchical clustering; red indicates high relative expression, and green indicates low relative expression. **a**, NFD vs. LFD; **b**, NFD vs. HFD; **c**, LFD vs. HFD. **d**-**f**, Differentially expressed lncRNAs in liver using Volcano plot. The red point in the plot represents the differentially expressed lncRNAs with statistical significance. **d**, NFD vs. LFD; **e**, NFD vs. HFD; **f**, LFD vs. HFD. NFD, normal fat diet group; HSF, high stearic acid diet group (C18:0/ C16:0 = 1:2); LSF, low stearic acid diet group (C18:0/ C16:0 = 1:8)

A total of 34,523 lncRNAs were analyzed. As shown by heat map and volcano plot, there were distinguishable gene expression profiles among the groups (Fig. 2). Compared with the NFD group, 258 lncRNAs were differentially expressed in the LSF group, including 152 that were upregulated and 106 that were downregulated (Fig. 2a, d), while 751 lncRNAs were differentially expressed in the HSF group, including 364 that were upregulated and 387 that were downregulated; more lncRNAs were differentially expressed in the HSF group than in the LSF group (Fig. 2b, e). Among these differentially expressed lncRNAs in comparison with the NFD group, there were 148 of the same lncRNAs in both the LSF and HSF groups. There were 216 differentially expressed lncRNAs between the LSF and HSF groups, including 116 that were upregulated and 100 that were downregulated (Fig. 2c, f).

### Differentially expressed microRNAs in the liver among groups

**Fig. 3:**
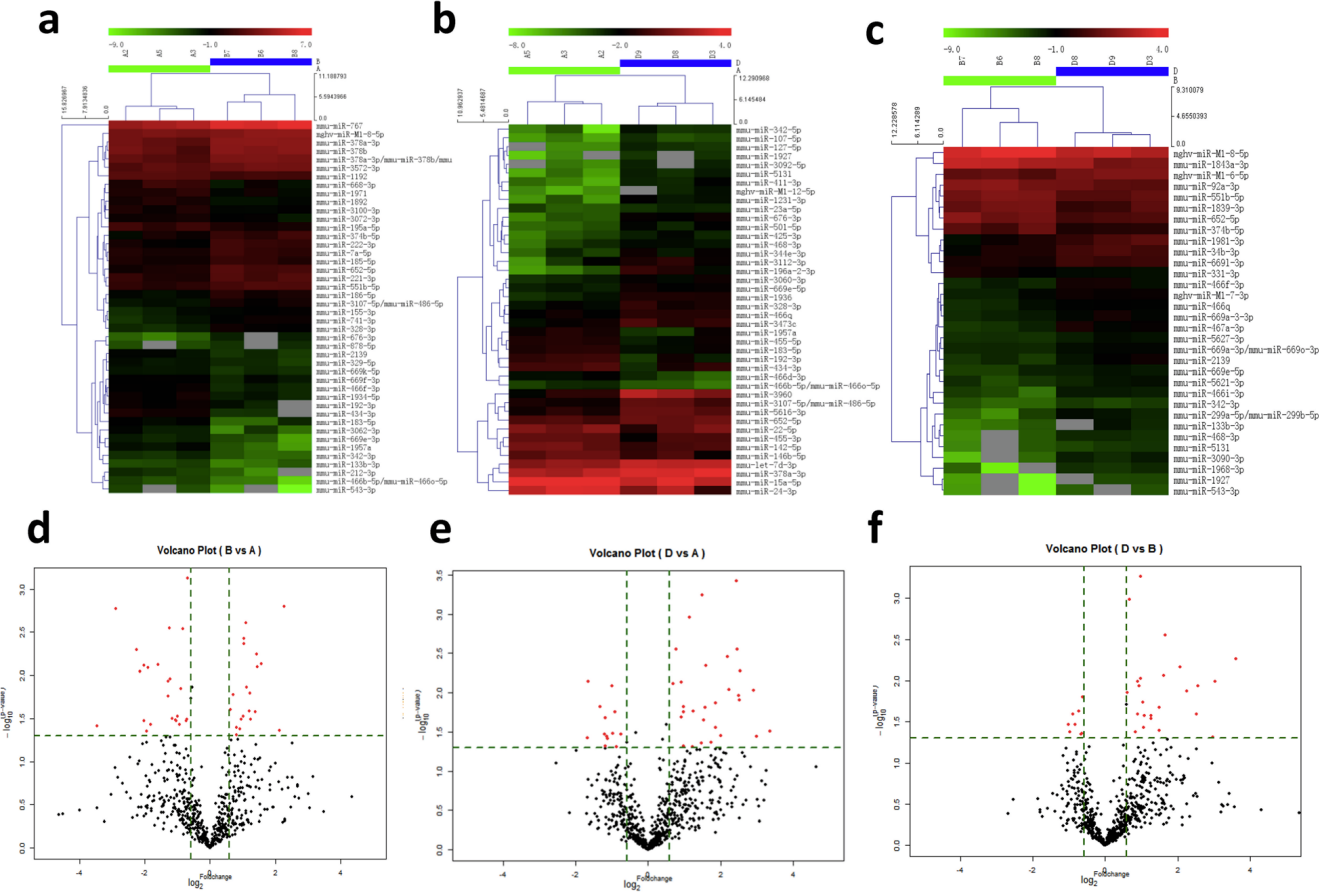
Differentially expressed mRNAs in liver of mice. **a**-**c**, Differentially expressed mRNAs in liver analyzed using hierarchical clustering; red indicates high relative expression, and green indicates low relative expression. **a**, NFD vs. LFD; **b**, NFD vs. HFD; **c**, LFD vs. HFD. **d**-**f**, Differentially expressed mRNAs in liver by Volcano plot. The red point in the plot represents the differentially expressed mRNAs with statistical significance. **d**, NFD vs. LFD; **e**, NFD vs. HFD; **f**, LFD vs. HFD. NFD, normal fat diet group; HSF, high stearic acid diet group (C18:0/ C16:0 = 1:2); LSF, low stearic acid diet group (C18:0/ C16:0 = 1:8)

A total of 3544 microRNAs were analyzed. Compared with the NFD group, 44 microRNAs were differentially expressed in the LSF group, including 20 that were upregulated and 24 that were downregulated (Fig. 3a, d), while 42 microRNAs were differentially expressed in the HSF group, including 29 that were upregulated and 13 that were downregulated (Fig. 3b, e). Among these differentially expressed microRNAs in comparison with the NFD group, there were 10 of the same microRNAs in both the LSF and HSF groups. There were 32 differentially expressed microRNAs between the LSF and HSF groups, including 24 that were upregulated and 8 that were downregulated (Fig. 3c, f).

### Differentially expressed mRNAs in the liver among groups

**Fig. 4:**
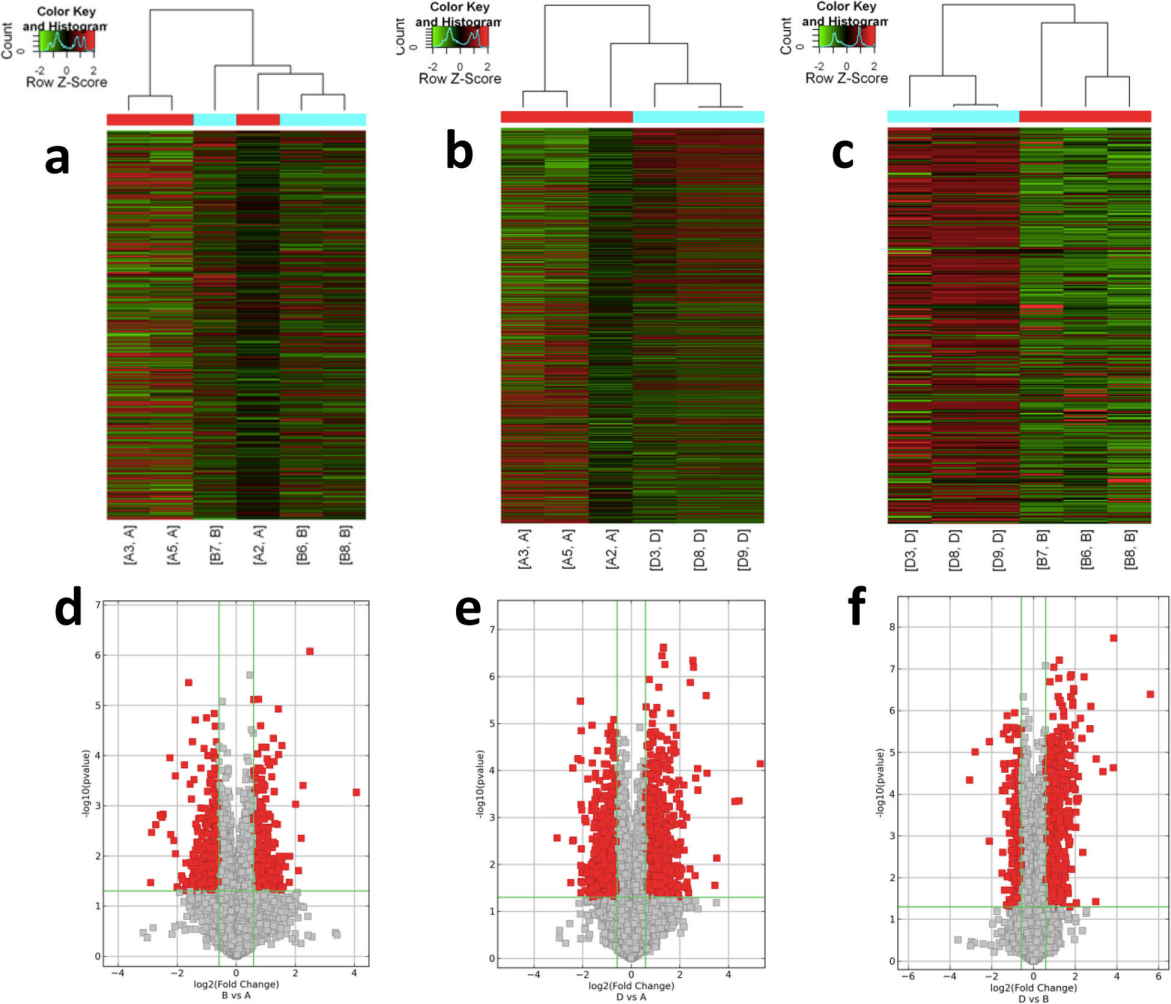
Differentially expressed microRNAs in liver tissues. **a**-**c**, Differentially expressed microRNAs in liver analyzed using hierarchical clustering; red indicates high relative expression, and green indicates low relative expression. **a**, NFD vs. LFD; **b**, NFD vs. HFD; **c**, LFD vs. HFD. **d**-**f**, Differentially expressed microRNAs in liver by Volcano plot. The red point in the plot represents the differentially expressed microRNAs with statistical significance. **d**, NFD vs. LFD; **e**, NFD vs. HFD; **f**, LFD vs. HFD. NFD, normal fat diet group; HSF, high stearic acid diet group (C18:0/ C16:0 = 1:2); LSF, low stearic acid diet group (C18:0/ C16:0 = 1:8)

Among 23,047 mRNAs, a total of 302 differentially expressed mRNAs were identified between the LSF group and the NFD group, including 140 that were upregulated and 162 that were downregulated (Fig. 4a, c), while 808 differentially expressed mRNAs were identified between the HSF group and the NFD group, including 433 that were upregulated and 375 that were downregulated (Fig. 4b, e). There were many more differentially expressed mRNAs in the HSF group than in the LSF group when compared with the NFD group. Additionally, among these differentially expressed mRNAs, 150 of the same mRNAs were differentially expressed in both the LSF and the HSF groups. There were 275 differentially expressed mRNAs between the LSF and HSF groups, including 230 that were upregulated and 45 that were downregulated (Fig. 4c, f).

### Functional analysis of differentially expressed mRNAs in the liver among groups

**Fig. 5:**
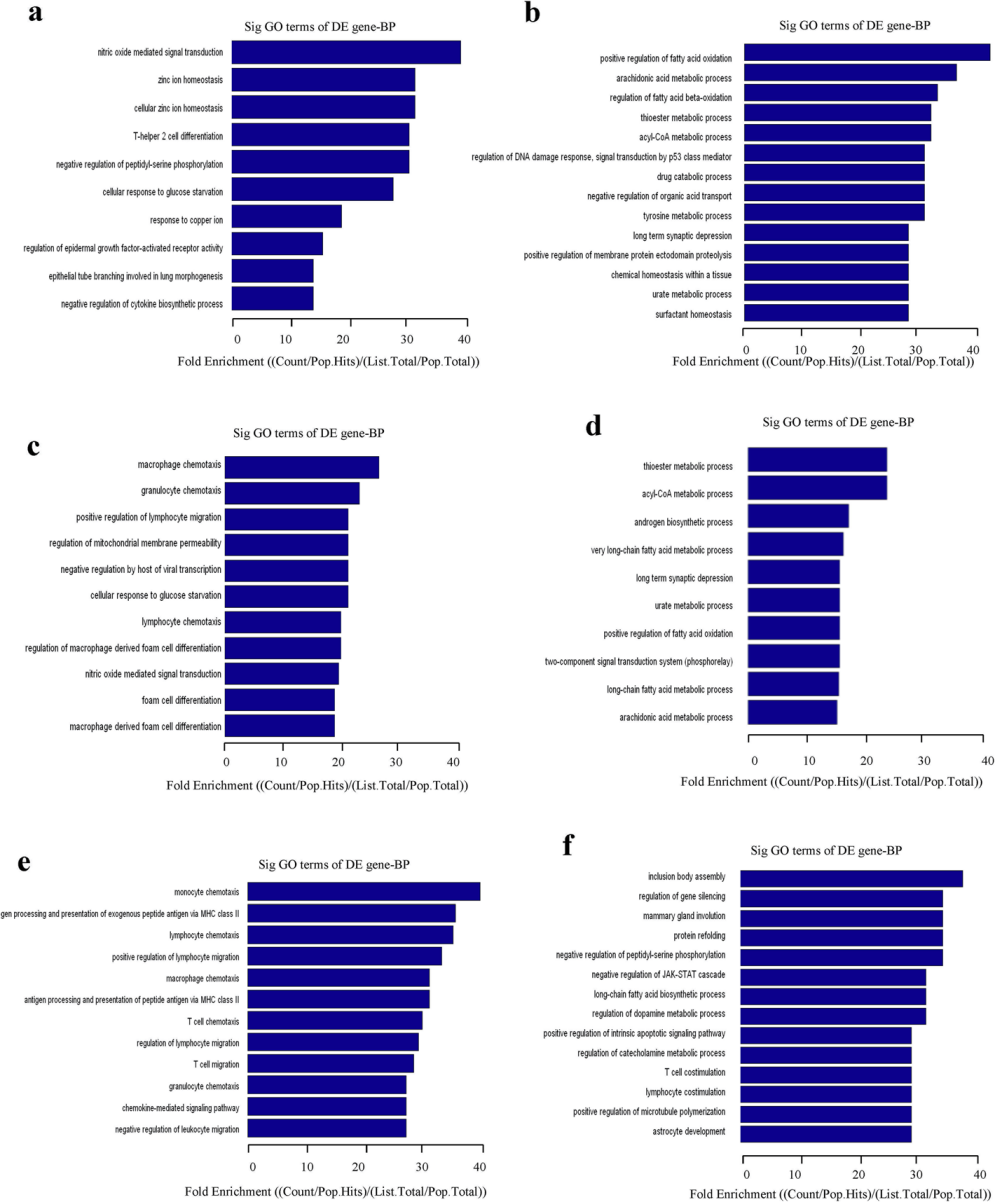
GO analyses of biological process for the differentially expressed mRNAs. **a**, **c** and **e**, Top ten fold enrichment terms of biological processes for mRNAs down-regulated. **a**, NFD vs. LFD; **c**, NFD vs. HFD; **e**, LFD vs. HFD. **b**, **d** and **f**, Top ten fold enrichment terms of biological processes for mRNAs up-regulated. **b**, NFD vs. LFD; **d**, NFD vs. HFD; **f**, LFD vs. HFD. The bar plot shows the top ten fold enrichment value of the significant enrichment terms

The GO analysis covered three domains: biological process (Fig. 5), cellular component (Fig. S4), and molecular function (Fig. S5), and this study mainly focused on biological processes. Compared with the NFD group, differentially upregulated mRNAs were involved in biological processes including nitric oxide-mediated signal transduction and cellular response to glucose starvation (Fig. 5a, c), and differentially downregulated mRNAs were involved in positive regulation of fatty acid oxidation, arachidonic acid metabolic process, thioester metabolic process, acyl-CoA metabolic process, long term synaptic depression, and urate metabolic process in both the LSF group and the HSD group (Fig. 5b, d). Compared with the LSF group, differentially upregulated mRNAs were involved in inclusion body assembly, regulation of gene silencing, and mammary gland involution (Fig. 5e), and differentially downregulated mRNAs were involved in monocyte chemotaxis, antigen processing and presentation of exogenous peptide antigen via MHC class II, and lymphocyte chemotaxis in the HSF group (Fig. 5f).

**Fig. 6:**
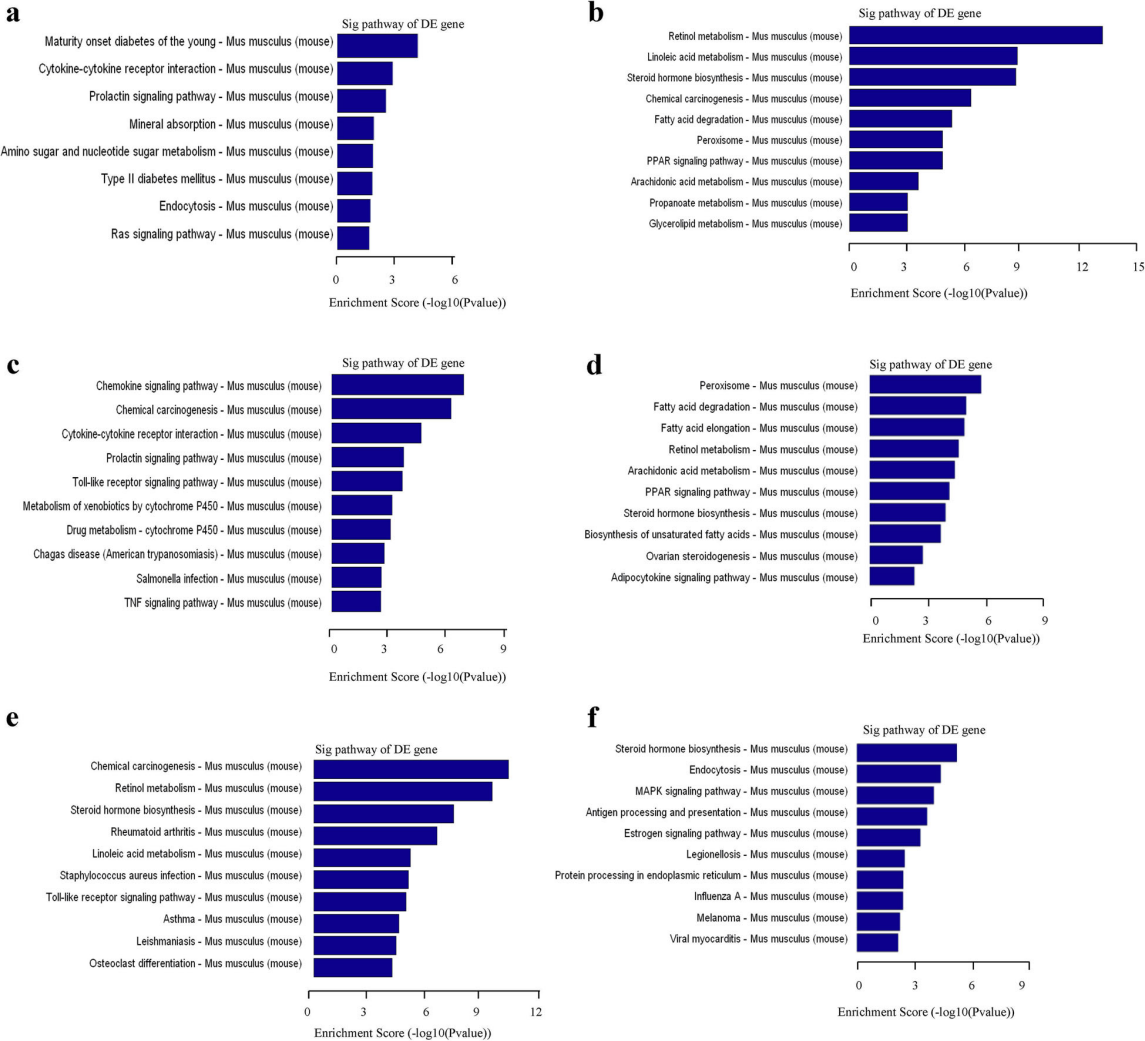
Pathway analyses of the differentially expressed mRNAs. **a**, **c** and **e**, Top ten score enrichment terms of pathways for mRNAs down-regulated. **a**, NFD vs. LFD; **c**, NFD vs. HFD; **e**, LFD vs. HFD. **b**, **d** and **f**, Top ten score enrichment terms of pathways for mRNAs up-regulated. **b**, NFD vs. LFD; **d**, NFD vs. HFD; **f**, LFD vs. HFD. The bar plot shows the top ten Enrichment score value of the significant enrichment pathways

Compared with the NFD group, the common pathways that the up-regulated transcripts involved in included cytokine-cytokine receptor intervention and the prolactin signaling pathway (Fig. 6a, c), and the common pathways that the downregulated transcripts involved in included retinol metabolism, fatty acid degradation, the peroxisome, steroid hormone biosynthesis, the PPAR signaling pathway, and arachidonic acid metabolism in both the LSF group and the HSD group (Fig. 6b, d). When comparing the differences between the LSF group and the HSD group, the enrichment pathways with the top 3 scores were chemical carcinogenesis, retinol metabolism, and steroid hormone biosynthesis for upregulated mRNAs (Fig. 6e) and steroid hormone biosynthesis, endocytosis and MAPK signaling pathway for downregulated mRNAs (Fig. 6f).

## Discussion

In the present study, the changes in metabolic and transcriptomic profiles between two high fat diet groups with different C18:0/C16:0 ratios have been compared in mice for the first time. The results showed that glucose and lipid metabolic disorders and inflammation were much more severe in HSF mice than in LSF mice. Compared with the NFD group, there were many more differentially expressed lncRNAs and mRNAs in HSF mice than in LSF mice, with distinguishable lncRNA, microRNA, and mRNA expression profiles between these two groups. The differentially expressed lncRNAs, microRNAs, and mRNAs between the LSF and HSF groups were much more numerous than those with the same changing trends in both the LSF and HSF groups, when compared with the NFD group. Additionally, some biological functions and pathways, except for energy metabolism regulation, were identified that the differentially expressed mRNAs between the HSF group and the LSF group were probably involved in C18:0/C16:0 ratio and obesity.

Many studies have shown that a high fat diet induces passive overfeeding, which leads to obesity and poor lipid profiles [22–24]. However, limited studies have focused on the effect of saturated fatty acid composition in diets, especially regarding the effect of the C18:0/C16:0 ratio on metabolism [25]. In the present study, compared with NFD mice, mice in both high fat diet groups (HSF and LSF) developed obesity. However, the body weight and body fat ratio of mice in the HSF group were much higher than those of mice in the LSF group. Additionally, there were more severe mitochondrial injuries in the liver and pancreas in the HSF group than in the LSF group. These may be explained by the decrease in fat oxidation in the HSF group when compared with the LSF group, as observed with an energy metabolism monitor. Additionally, previous studies support this finding that saturated fatty acids with longer chain lengths have slower oxidation rates [14, 15]. These results suggest that obesity induced by high fat diets is associated with the chain length of the saturated fatty acids in diets, and a high C18:0 diet is more likely to lead to obesity than an isocaloric high C16:0 diet C18:0/C16:0 ratio and insulin resistance.

Increased circulating FFAs, especially SFAs, are key factors that cause insulin resistance in obesity [26]. C16:0 and C18:0 are the most common long chain saturated fatty acids in food and the human body, and they are most closely related to insulin resistance and type 2 diabetes [19, 27]. Human studies have revealed that circulating SFAs, especially C16:0 and C18:0, were associated with higher diabetes risk [28, 29]. However, whether there is any difference between C16:0 and C18:0, especially from diet, in terms of their effect on insulin resistance and diabetes has not been reported in humans. A study from van den Berg SA demonstrated that a high fat diet rich in stearate induced a metabolic state favoring low oxidative metabolism and whole-body insulin resistance characterized by severe hepatic insulin resistance [11]. However, the circulation and organism FFA levels were not detected. A previous study found that C18:0 exhibited a stronger lipotoxic role than C16:0 in both mouse islets and rat insulinoma INS-1 cells [30]. In the present study, the high fat diet with high C18:0 induced more severe insulin resistance in mice than the isocaloric high C16:0 diet, in accordance with previous studies. In addition, the levels of FFAs, including C16:0 and C18:0, in serum and liver were much higher in the HSF group than those in the LSF group. The high fat diet rich in C18:0 led to a higher level of C18:0 in the body in the HSF group. This resulted in more intermediate lipid metabolites due to a relatively lower capacity of C18:0 in oxidation and incorporation into triacylglycerol. Moreover, the higher level of C18:0 may inhibit the conversion of C18:0 from C16:0 in the body, leading to the accumulation of C16:0 in the HSF group, as higher serum and liver C16:0 levels in the HSF group than in the LSF group were observed. These probably collectively contribute to the severe insulin resistance in the HSF group.

C18:0/C16:0 ratio and differences in regulation of biological processes and signaling pathways.

Fatty acids, not only an energy resource but also an important messenger or signaling molecules, are involved in multiple pathophysiological processes in the body [31]. Gene microarray analysis of lncRNA, microRNA and mRNA profiles was carried out to further explore whether high fat diets with different C18:0/C16:0 ratios result in different changes in biological processes and to determine which signaling pathways are affected. As expected, mice in both HSF group and LSF group exhibited distinguishable gene expression profiles, compared with the NFD mice. Additionally, more differentially expressed lncRNAs and mRNAs were observed in the HSF group than those in the LSF group, indicating that high C18:0 probably leads to changes in more biological processes or signaling pathways. In addition, the differentially expressed lncRNAs, microRNAs, and mRNAs between the LSF and HSF groups were much more numerous than those with the same changing trends in both the LSF and HSF groups, when compared with the NFD group. This suggests that there may be more differences between C16:0 and C18:0 than similarities in their biological functions or mechanisms, which has been less of a focus so far. The results from GO analysis indicated that C16:0 and C18:0 were involved in some of the same biological processes, including cellular response to glucose starvation, positive regulation of fatty acid oxidation, arachidonic acid metabolic process, thioester metabolic process, long term synaptic depression and urate metabolic process. Most of these biological processes have been widely explored in studies on fatty acids metabolism and function [32–34]. Further pathway analysis showed that the common pathways possibly regulated by C16:0 and C18:0 include cytokine-cytokine receptor intervention, prolactin signaling pathway, retinol metabolism, fatty acid degradation, peroxisome, steroid hormone biosynthesis, PPAR signaling pathway, and arachidonic acid metabolism. These pathways have been found to be mostly involved in glucose and lipid metabolism [35–38], in accordance with the common biological processes found between the LSF group and the HSD group. These results indicate that the similarity in biological functions and mechanisms between C16:0 and C18:0 are mainly in relation to glucose and lipid metabolism, in which they act as substrates in metabolism, and the chain length is probably a key factor in determining their effects. It is noteworthy that GO analysis showed significantly different effects between C16:0 and C18:0 in many biological processes other than glucose and lipid metabolism, including inclusion body assembly, regulation of gene silencing, mammary gland involution, monocyte chemotaxis, antigen processing and presentation of exogenous peptide antigen via MHC class II, and lymphocyte chemotaxis. Studies about the effects of specific fatty acids on these biological processes are very limited so far, especially for C16:0 and C18:0. For the different pathways that the differentially expressed mRNAs regulated, the score enrichment pathways included chemical carcinogenesis, retinol metabolism, steroid hormone biosynthesis, endocytosis and MAPK signaling. These signaling pathways have been found to be mostly involved in the regulation of immunological processes [39–42], which was consistent with the GO analysis results. In these biological processes and pathways, C16:0 and C18:0 probably act as signaling molecules. Several studies have reported a high fat diet regulated immune function [43, 44]. However, the pathways for immunological regulation by C18:0 and C16:0 have seldom been reported. Most of the biological processes and pathways observed in the present study have not been investigated. It is worthwhile to further investigate the differentially expressed lncRNAs, microRNAs and mRNAs and their interactions between the two high fat diet groups in order to highlight the different biological functions and related mechanisms between C18:0 and C16:0.

For such complex data, two aspects of analysis are suggested. First, the different biological functions between C16:0 and C18:0 can be focused on. After the differentially expressed lncRNAs of HSF vs. NFD, LSF vs. NFD, and HSF vs. LSF have been found in the present study, the overlapping lncRNAs of these three comparisons should be further studied. These overlapping lncRNAs, their predicted target genes and related biological processes or pathways are probably responsible for the differences between C16:0 and C18:0, providing a clue for further detailed and deeper research, especially on lncRNA-related mechanisms. Then, one or some specific lncRNAs of interest can also be focused on. The same approach is taken for microRNAs and mRNAs in the analysis of differences between C16:0 and C18:0. Based on these analyses, interaction analysis among these overlapping lncRNAs, microRNAs, and mRNAs can be performed to explore a more complex mechanism, such as the classic negative regulation of miRNAs by lncRNAs, acting as miRNA sponges. Based on the present results, the different biological functions between C16:0 and C18:0 are probably mainly in the context of immunological processes. Second, the common new biological functions of C16:0 and C18:0 can also be focused on. After the differentially expressed lncRNAs, microRNAs, and mRNAs of HSF vs. NFD, and LSF vs. NFD have been found, the overlapping genes and predicted biological processes or pathways can provide clues for deeper mechanistic studies for specific lncRNAs, microRNAs, mRNAs and their interactions. Based on the present results, the common biological functions between C16:0 and C18:0 are mainly involved in the regulation of glucose and lipid metabolism, which need to be deeply explored for new detailed mechanisms.

### Study strengths and limitations

This study provided a detailed base data at metabolic and gene transcriptional levels, and suggested more widely different biological functions between C18:0 and C16:0 other than energy metabolism. One limitation of this study was that only a preliminary bioinformatics analysis was performed. There was a lack of validation results at the cellular and molecular levels. The existence of possible miss-distance effect and non-specific effect cannot be excluded in the present study. In addition, specific interactions among mRNAs, microRNAs and lncRNAs were not deeply explored. Further studies should be carried out based on the present data to clarify related mechanisms.

## Conclusions

A high fat diet with a high C18:0/C16:0 ratio induces a worse lipid profile, severe insulin resistance and affects more lncRNA and mRNA expression than an isocaloric low C18:0/C16:0 ratio diet in mice. These results provide new insights into the different biological functions and related mechanisms between C16:0 and C18:0. The ratio of C18:0/C16:0 in diets should be taken into account in practice for accurate nutrition and health in future.

## Supplementary information

**Additional file 1: Supplementary Methods**-GC-MS conditions and method performance. **Table S1.** Composition of diets (g/1000 g diets). **Table S2**. The changes in the body weight in mice. **Table S3**. Comparisons of body composition in mice. **Table S4**. The total body fat ratio and liver fat ratio in mice. **Table S5**. The levels of fasting serum indices in mice. **Table S6.** Liver fatty acids profile in mice. **Figure S1.** Indirect calorimetry of mice for consecutive 72 h. a, Energy expenditure. b, Carbohydrate oxidation. c, Fatty acid oxidation. d, Respiratory exchange rates. NFD, normal fat diet group; HSF, high stearic acid diet group (C18:0/ C16:0 = 1:2); LSF, low stearic acid diet group (C18:0/ C16:0 = 1:8). EE, energy expenditure. RER, respiratory exchange rate. *N* = 6 for each group. ^*^P < 0.05, compared with NFD group; ^*#*^*P* < 0.05, compared wtih LSF group. **Figure S2.** Oral glucose tolerance test of mice. NFD, normal fat diet group; HSF, high stearic acid diet group (C18:0/ C16:0 = 1:2); LSF, low stearic acid diet group (C18:0/ C16:0 = 1:8). *N* = 10 for each group. ^*^P < 0.05, compared with NFD group at the same time point; ^*#*^*P* < 0.05, compared wtih LSF group at the same time point. NFD, normal fat diet group; HSF, high stearic acid diet group (C18:0/ C16:0 = 1:2); LSF, low stearic acid diet group (C18:0/ C16:0 = 1:8). **Figure S3.** Detection of cell ultra-structures in liver and pancreas by transmission electron microscopy. a-c, Changes in mitochondria in liver; d-f, Changes in insulin granules in pancreas. a and d, normal fat diet group; b and e, low stearic acid diet group (C18:0/ C16:0 = 1:8); c and f, high stearic acid diet group (C18:0/ C16:0 = 1:2). **Figure S4.** GO analyses of cellular component for the differentially expressed mRNAs in mice. a, c and e, Top ten fold enrichment terms of cellular components for mRNAs down-regulated. a, NFD vs. LFD; c, NFD vs. HFD; e, LFD vs. HFD. b, d and f, Top ten fold enrichment terms of cellular components for mRNAs up-regulated. b, NFD vs. LFD; d, NFD vs. HFD; f, LFD vs. HFD. The bar plot shows the top ten fold enrichment value of the significant enrichment terms. **Figure S5.** GO analyses of molecular function for the differentially expressed mRNAs in mice. a, c and e, Top ten fold enrichment terms of molecular functions for mRNAs down-regulated. a, NFD vs. LFD; c, NFD vs. HFD; e, LFD vs. HFD. b, d and f, Top ten fold enrichment terms of molecular functions for mRNAs up-regulated. b, NFD vs. LFD; d, NFD vs. HFD; f, LFD vs. HFD. The bar plot shows the top ten fold enrichment value of the significant enrichment terms.

## Data Availability

The datasets generated during and/or analyzed during the current study are available from the corresponding author on reasonable request.

## Abbreviations

C16:0: Palmitic acid
C18:0: Stearic acid
EE: Energy expenditure
FFAs: Free fatty acids
GO analysis: Gene Ontology analysis
HDL-c: High density lipoprotein cholesterol
HOMA-IR: Homeostasis model assessment of insulin resistance
HSF: High fat diet with high C18:0 / C16:0 ratio
IL-6: Interleukin-6
LDL-c: Low density lipoprotein cholesterol
LSF: High fat diet with a low C18:0 / C16:0 ratio
NFD: Normal fat diet
OGTT: Oral glucose tolerance test
RER: Respiratory exchange rate
SFAs: Saturated fatty acids
TC: Total cholesterol
TG: Triglyceride
TNF-α: Tumor necrosis factor-α
