# Supplementary material for: A high fat diet with a high C18:0/C16:0 ratio induced worse metabolic and transcriptomic profiles in C57BL/6 mice
Source: Lipids Health Dis. 2020 Jul 21;19:172. doi: 10.1186/s12944-020-01346-z (PMC7372854; doi:10.1186/s12944-020-01346-z)
Supplement: Supplementary file 1 — Additional file 1: Supplementary Methods-GC-MS conditions and method performance. Table S1. Composition of diets (g/1000 g diets). Table S2. The changes in the body weight in mice. Table S3. Comparisons of body composition in mice. Table S4. The total body fat ratio and liver fat ratio in mice. Table S5. The levels of fasting serum indices in mice. Table S6. Liver fatty acids profile in mice. Figure S1. Indirect calorimetry of mice for consecutive 72 h. a, Energy expenditure. b, Carbohydrate oxidation. c, Fatty acid oxidation. d, Respiratory exchange rates. NFD, normal fat diet group; HSF, high stearic acid diet group (C18:0/ C16:0 = 1:2); LSF, low stearic acid diet group (C18:0/ C16:0 = 1:8). EE, energy expenditure. RER, respiratory exchange rate. N = 6 for each group. *P < 0.05, compared with NFD group; #P < 0.05, compared wtih LSF group. Figure S2. Oral glucose tolerance test of mice. NFD, normal fat diet group; HSF, high stearic acid diet group (C18:0/ C16:0 = 1:2); LSF, low stearic acid diet group (C18:0/ C16:0 = 1:8). N = 10 for each group. *P < 0.05, compared with NFD group at the same time point; #P < 0.05, compared wtih LSF group at the same time point. NFD, normal fat diet group; HSF, high stearic acid diet group (C18:0/ C16:0 = 1:2); LSF, low stearic acid diet group (C18:0/ C16:0 = 1:8). Figure S3. Detection of cell ultra-structures in liver and pancreas by transmission electron microscopy. a-c, Changes in mitochondria in liver; d-f, Changes in insulin granules in pancreas. a and d, normal fat diet group; b and e, low stearic acid diet group (C18:0/ C16:0 = 1:8); c and f, high stearic acid diet group (C18:0/ C16:0 = 1:2). Figure S4. GO analyses of cellular component for the differentially expressed mRNAs in mice. a, c and e, Top ten fold enrichment terms of cellular components for mRNAs down-regulated. a, NFD vs. LFD; c, NFD vs. HFD; e, LFD vs. HFD. b, d and f, Top ten fold enrichment terms of cellular components for mRNAs up-regulated. b, NFD vs. LFD; d, [file 12944_2020_1346_MOESM1_ESM.docx]

**Supplementary methods-GC-MS conditions as well as method performance**

**1. Standard solutions preparation and method validation**

Stock solutions of 11 fatty acids and internal standards (heptadecanoic acid) were prepared at 1000.00 μg/mL in methanol. Working solutions were prepared with methanol at concentrations of 1.10–1000.00 μg/mL. All standard solutions were stored at −20℃ until they were required. Calibration samples were prepared by spiking with 11 different concentrations of fatty acids standards. Limit of detection (LOD) were defined as lowest concentrations with signal-to-noise (S/N) ratios of 10. Repeatability of calibration samples was expressed as coefficients of variation (CV%) and percentage biases (bias%), respectively. The CV was 2.97%-7.73% and the bias was 83.40%-103.80%.

**2. Sample preparation**

Briefly, aliquots (200 μL) of serum were spiked with internal standard (I.S.) working solution (200 μL heptadecanoic acid C17:0 200 μg/mL), and 1 mL 0.05% H_2_SO_4_ was then added to deposit protein. The FFA was extracted using 3 mL ethyl acetate and shaking with a vortex mixer for 60 s, and then centrifuged at 4,000 × g for 10 min at room temperature. The ethyl acetate phase was evaporated to dryness under N_2_. Following the addition of 2 mL 10% H_2_SO_4_–CH_3_OH and incubation in a 62℃ water bath for 2 h, 2 mL saturated sodium chloride and 2 mL hexane were sequentially added and mixed for 60s to obtain the fatty acid methyl esters. Samples were evaporated to dryness under N_2_ gas, and 100 μL hexane was added to each tube prior to analysis

**3. Gas chromatography–mass spectrometry**

GC–MS analysis was performed using a TRACE gas chromatograph with a Polaris Q mass spectrometer (Thermo Finnigan, Austin, TX, USA). Helium was used as the carrier gas. A split injector (the split ratio being 1:10) at 230℃ was used to add the sample (1.0 μL) onto a J&W DB-WAX (30 m × 0.25 mm I.D., 0.25 mm film thickness) capillary column. Fatty acid methyl esters were separated at constant flow with the following oven program: (a) initially 50℃ for 2 min; (b) increased temperature at a rate of 10℃/min up to 200℃; (c) maintained at 200℃ for 10 min; (d) increased temperature at a rate of 10℃/min up to 220◦C; (e) maintained at 220℃ for 15 min. The transfer line was maintained at 230℃. The ion trap mass spectrometer was operated under electron bomb ionization (EI) mode. Mass spectra of m/z 30–450 were collected by full scan mode with 0.58 s/scan velocity. Solvent delay time was 5 min. The source temperature was 230℃ with the electron energy at 70 eV.

**Supplementary methods-Microarrays methods**

**1. lncRNA and mRNA microarrarys**

**RNA extraction**

Total RNA was isolated by using TRIzol (Invitrogen) and miRNeasy mini kit (QIAGEN) according to the manufacturer’s instructions, which efficiently recovered all RNA species. RNA quality and quantity were measured by using nanodrop spectrophotometer (ND-1000, Nanodrop Technologies) and RNA Integrity was determined by gel electrophoresis.

**DNA microarray**

The Arraystar Mouse LncRNA Array v2.0 was designed for researchers who had interests in profiling both LncRNAs and protein-coding RNAs in mouse genome. 31,423 LncRNAs were collected from the authoritative data sources including RefSeq, UCSC Knowngenes, Ensembl and many related literatures.

**RNA labeling and array hybridization**

Sample labeling and array hybridization were performed according to the Agilent One-Color Microarray-Based Gene Expression Analysis protocol (Agilent Technology) with minor modifications. Briefly, mRNA was purified from total RNA after removal of rRNA (mRNA-ONLY™ Eukaryotic mRNA Isolation Kit, Epicentre). Then, each sample was amplified and transcribed into fluorescent cRNA along the entire length of the transcripts without 3’ bias utilizing a random priming method. The labeled cRNAs were purified by RNeasy Mini Kit (Qiagen). The concentration and specific activity of the labeled cRNAs (pmol Cy3/μg cRNA) were measured by NanoDrop ND-1000. 1 μg of each labeled cRNA was fragmented by adding 5 μl 10 × Blocking Agent and 1 μl of 25 × Fragmentation Buffer, then heated the mixture at 60 °C for 30 min, finally 25 μl 2 × GE Hybridization buffer was added to dilute the labeled cRNA. 50 μl of hybridization solution was dispensed into the gasket slide and assembled to the LncRNA expression microarray slide. The slides were incubated for 17 hours at 65°C in an Agilent Hybridization Oven. The hybridized arrays were washed, fixed and scanned with using the Agilent DNA Microarray Scanner (part number G2505C).

**Data analysis**

Agilent Feature Extraction software (version 11.0.1.1) was used to analyze acquired array images. Quantile normalization and subsequent data processing were performed using the GeneSpring GX v11.5.1 software package (Agilent Technologies). After quantile normalization of the raw data, LncRNAs and mRNAs that at least 6 out of 9 samples had flags in Present or Marginal (“All Targets Value”) were chosen for further data analysis. Differentially expressed LncRNAs/mRNAs were identified through Fold Change filtering. Hierarchical Clustering was performed using the Agilent GeneSpring GX software (version 11.5.1). GO analysis and Pathway analysis were performed in the standard enrichment computation method.

**2. miRNA microarrays methods**

**RNA extraction**

Total RNA was isolated using TRIzol (Invitrogen) and miRNeasy mini kit (QIAGEN) according to the manufacturer’s instructions, which efficiently recovered all RNA species, including miRNAs. RNA quality and quantity were measured by using nanodrop spectrophotometer (ND-1000, Nanodrop Technologies) and RNA Integrity was determined by gel electrophoresis.

**miRNA microarray**

The 7th generation of miRCURYTM LNA Array (v.18.0) (Exiqon) contains 3100 capture probes, covering all human, mouse and rat microRNAs annotated in miRBase 18.0, as well as all viral microRNAs related to these species. In addition, this array contains capture probes for 25 miRPlus™ human microRNAs.

**RNA labeling**

After RNA isolation from the samples, the miRCURY™ Hy3™/Hy5™ Power labeling kit (Exiqon, Vedbaek, Denmark) was used according to the manufacturer’s guideline for miRNA labelling. One microgram of each sample was 3'-end-labeled with Hy3TM fluorescent label, using T4 RNA ligase by the following procedures: RNA in 2.0 μL of water was combined with 1.0 μL of CIP buffer and CIP (Exiqon). The mixture was incubated for 30 min at 37°C, and was terminated by incubation for 5 min at 95°C. Then 3.0 μL of labeling buffer, 1.5 μL of fluorescent label (Hy3TM), 2.0 μL of DMSO, 2.0 μL of labeling enzyme were added into the mixture. The labeling reaction was incubated for 1 h at 16°C, and terminated by incubation for 15 min at 65°C.

**Array hybridization**

After stopping the labeling procedure, the Hy3TM-labeled samples were hybridized on the miRCURYTM LNA Array (v.18.0) (Exiqon) according to the array manual. The total 25 μL mixture from Hy3TM-labeled samples with 25 μL hybridization buffer was first denatured for 2 min at 95°C, incubated on ice for 2 min and then hybridized to the microarray for 16–20 h at 56°C in a 12-Bay Hybridization Systems (Hybridization System - Nimblegen Systems, Inc., Madison, WI, USA), which provided an active mixing action and constant incubation temperature to improve hybridization uniformity and enhance signal. Following hybridization, the slides were achieved, washed several times using Wash buffer kit (Exiqon), and finally dried by centrifugation for 5 min at 400 rpm. Then the slides were scanned using the Axon GenePix 4000B microarray scanner (Axon Instruments, Foster City, CA).

**Data analysis**

Scanned images were then imported into GenePix Pro 6.0 software (Axon) for grid alignment and data extraction. Replicated miRNAs were averaged and miRNAs that intensities>=30 in all samples were chosen for calculating normalization factor. Expressed data were normalized using the Median normalization. After normalization, significant differentially expressed miRNAs between two groups were identified through Volcano Plot filtering. Hierarchical clustering was performed using MEV software (v4.6, TIGR).

**Table S1** **Composition of diets (g/1000g diets)**

| Ingredients | ND | HSF | LSF |
| --- | --- | --- | --- |
| Casein | 200 | 200 | 200 |
| L-Cystine | 3 | 3 | 3 |
| Corn Starch | 397.485 | 287.485 | 287.485 |
| Maltodextrin | 132 | 132 | 132 |
| Sucrose | 100 | 100 | 100 |
| Cellulose | 50 | 50 | 50 |
| Soybean Oil | 70 | 30 | 30 |
| Palm oil | 0 | 0 | 150 |
| Lard | 0 | 150 | 0 |
| Mineral Mix | 35 | 35 | 35 |
| Vitamin Mix | 10 | 10 | 10 |
| Choline Bitartrate | 2.5 | 2.5 | 2.5 |
| Antioxidant | 0.015 | 0.015 | 0.015 |
| Total amount | 1000 | 1000 | 1000 |
| Total energy (kJ) | 16558.86 | 18841.34 | 18841.34 |
| Energy from fat (%) | 15 | 36 | 36 |

**Table S2. The changes in the body weight in mice**

| Weeks | NFD | LSF | HSF |
| --- | --- | --- | --- |
| 0 | 23.03±0.58 | 23.47±0.97 | 23.02±0.73 |
| 1 | 23.02±0.73 | 23.83±0.66 | 23.83±0.87 |
| 2 | 23.57±1.29 | 25.20±0.89 | 25.00±0.66 |
| 3 | 24.35±1.34 | 27.48±1.58^*^ | 26.73±1.57^*^ |
| 4 | 25.85±1.23 | 29.83±1.38^*^ | 29.43±2.43^*^ |
| 5 | 27.22±1.38 | 31.72±2.06^*^ | 32.68±2.29^*^ |
| 6 | 28.33±1.54 | 33.75±1.56^*^ | 35.43±2.44^*#^ |
| 7 | 29.62±1.49 | 35.13±1.46^*^ | 37.15±2.57^*#^ |
| 8 | 30.73±1.43 | 37.21±1.16^*^ | 38.95±1.96^*#^ |
| 9 | 32.10±1.73 | 38.65±0.90^*^ | 41.33±1.78^*#^ |
| 10 | 32.90±1.27 | 39.80±1.03^*^ | 42.09±1.41^*#^ |

All values are presented as mean ± SEM (n=10). NFD, normal fat diet group; HSF, high stearic acid diet group (C18:0/ C16:0=1:2); LSF, low stearic acid diet group (C18:0/ C16:0=1:8). ^*^ Compared with the NFD group, *P* < 0.05. ^#^ Compared with the LSF group, *P* < 0.05.

**Table S3 Comparisons of body composition in mice**

| Body composition (g) | NFD | LSF | HSF |
| --- | --- | --- | --- |
| Body weight | 33.34±0.82 | 40.45±1.07^*^ | 43.6±1.62^*#^ |
| Muscle mass | 11.38±0.52 | 13.11±0.48 | 13.55±0.45 |
| Fat mass | 1.19±0.45 | 9.13±0.46^*^ | 11.98±0.52^*#^ |
| Viceral fat mass | 0.53±0.06 | 4.23±0.08^*^ | 5.62±0.12^*#^ |
| Subcutaneous fat mass | 0.66±0.04 | 4.9±0.12^*^ | 6.35±0.48^*#^ |

All values are presented as mean ± SEM (n=10). NFD, normal fat diet group; HSF, high stearic acid diet group (C18:0/ C16:0=1:2); LSF, low stearic acid diet group (C18:0/ C16:0=1:8). ^*^ Compared with the NFD group, *P* < 0.05. ^#^ Compared with the LSF group, *P* < 0.05.

**Table S4 The total body fat ratio and liver fat ratio in mice**

| Fat ratio (%) | NFD | LSF | HSF |
| --- | --- | --- | --- |
| Total fat ratio | 9.45±0.41 | 41.05±0.52^*^ | 46.92±0.7^*#^ |
| Liver fat ratio | 9.38±0.35 | 15.87±0.56^*^ | 18.34±0.63^*#^ |

All values are presented as mean ± SEM (n=10). NFD, normal fat diet group; HSF, high stearic acid diet group (C18:0/ C16:0=1:2); LSF, low stearic acid diet group (C18:0/ C16:0=1:8). ^*^ Compared with the NFD group, *P* < 0.05. ^#^ Compared with the LSF group, *P* < 0.05.

**Table S5 The levels of fasting serum indices in mice**

| Indices | NFD | LSF | HSF |
| --- | --- | --- | --- |
| Glucose (mmol/L) | 4.5±0.46 | 7.24±1.19^*^ | 9.35±1.32^*#^ |
| Insulin (mmol/L) | 11.58±0.39 | 23.96±5.4^*^ | 33.54±1.8^*#^ |
| HOMA-IR | 2.27±0.22 | 6.2±0.1^*^ | 13.53±1.56^*#^ |
| TC (mmol/L) | 3.21±0.28 | 4.87±0.38^*^ | 4.91±0.28^*^ |
| TG (mmol/L) | 0.58±0.1 | 0.71±0.12^*^ | 0.8±0.14^*#^ |
| HDL-c (mmol/L) | 2.61±0.2 | 3.65±0.21^*^ | 3.43±0.25^*^ |
| LDL-c (mmol/L) | 0.26±0.08 | 0.64±0.11^*^ | 0.69±0.07^*^ |
| HDL/LDL | 10.59±0.76 | 5.83±0.75^*^ | 5.02±0.34^*#^ |
| TNF-α (pg/mL) | 1.75±0.23 | 2.75±0.11^*^ | 2.85±0.18^*^ |
| IL-6 (pg/mL) | 2.91±0.24 | 4.13±0.11^*^ | 4.48±0.17^*#^ |

All values are presented as mean ± SEM (n=10). All of the parameters were mesured and calculated in the fasting state. HOMA-IR, Homeostasis model assessment-insulin resistence; TC, total cholesterol; TG, triglyceride; HDL-c: High density lipoprotein cholesterol; LDL-c: Low density lipoprotein cholesterol; IL-6: Interleukin-6; TNF-α: Tumor necrosis factor-α; NFD, normal fat diet group; HSF, high stearic acid diet group (C18:0/ C16:0=1:2); LSF, low stearic acid diet group (C18:0/ C16:0=1:8). *^*^ P* < 0.05, compared with the NFD group; *^#^ P* < 0.05, compared with the LSF group.

**Table S6 Liver fatty acids profile in mice**

| Fatty acids (μg/mL) | NFD | LSF | HSF |
| --- | --- | --- | --- |
| C14:0 | 22.36±7.39 | 31.59±2.01 | 44.63±5.49 |
| C16:0 | 1260.45±141.31 | 2057.52±37.34^*^ | 2375.92±44.24^*#^ |
| C16:1 | 291.08±132.97 | 534.38±54.29^*^ | 615.8±113.6^*^ |
| C18:0 | 263.65±176.61 | 246.95±12.8^*^ | 422.27±56.22^*#^ |
| C18:1 | 924.93±272.74 | 2430.89±59.88^*^ | 3053.13±327.72^*#^ |
| C18:2 | 1705.99±246.38 | 1468.52±43.62^*^ | 1649.87±116.15 |
| γ- C18:3 | 117.19±18.11 | 130±32.39 | 138.43±31.64 |
| C18:3 | 1779.34±379.37 | 528.51±42.73^*^ | 1046.36±62.25^*#^ |
| C20:2 | 10.59±1.81 | 7.17±4.59 | 15.84±4.31 |
| C20:4 | 317.03±50.04 | 441.88±4.37^*^ | 394.99±54.89^*^ |
| C20:5 | 51.21±15.89 | 23.23±1.02 | 51.62±1.74 |
| saturated fatty acid | 1546.46±496.44 | 2336.06±50.13^*^ | 2842.82±6.48^*#^ |
| unsaturated fatty acid | 5197.36±1087.71 | 5564.58±103.18^*^ | 6966.05±179.44^*#^ |
| total free fatty acids | 6452.74±1431.13 | 7366.27±1.24^*^ | 9083.07±59.36^*#^ |

All values are presented as mean ± SEM (n=10). NFD, normal fat diet group; HSF, high stearic acid diet group (C18:0/ C16:0=1:2); LSF, low stearic acid diet group (C18:0/ C16:0=1:8). ^*^ Compared with the NFD group, *P* < 0.05. ^#^ Compared with the LSF group, *P* < 0.05.

Fig.S1


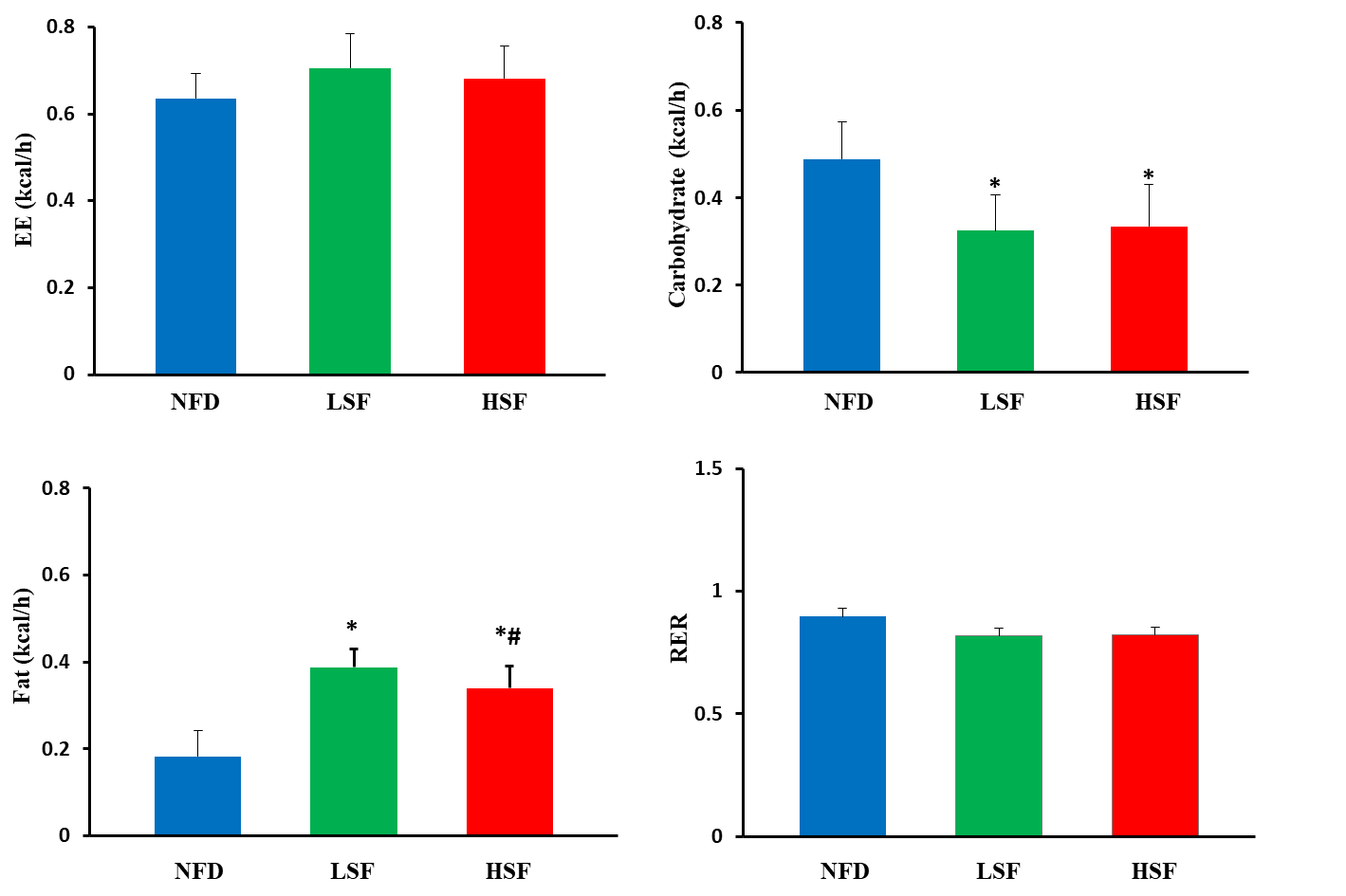


Fig. S2


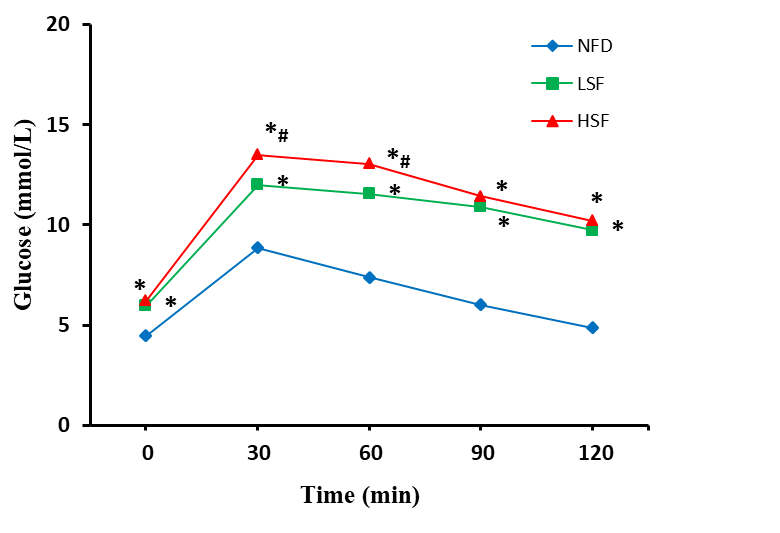


Fig. S3


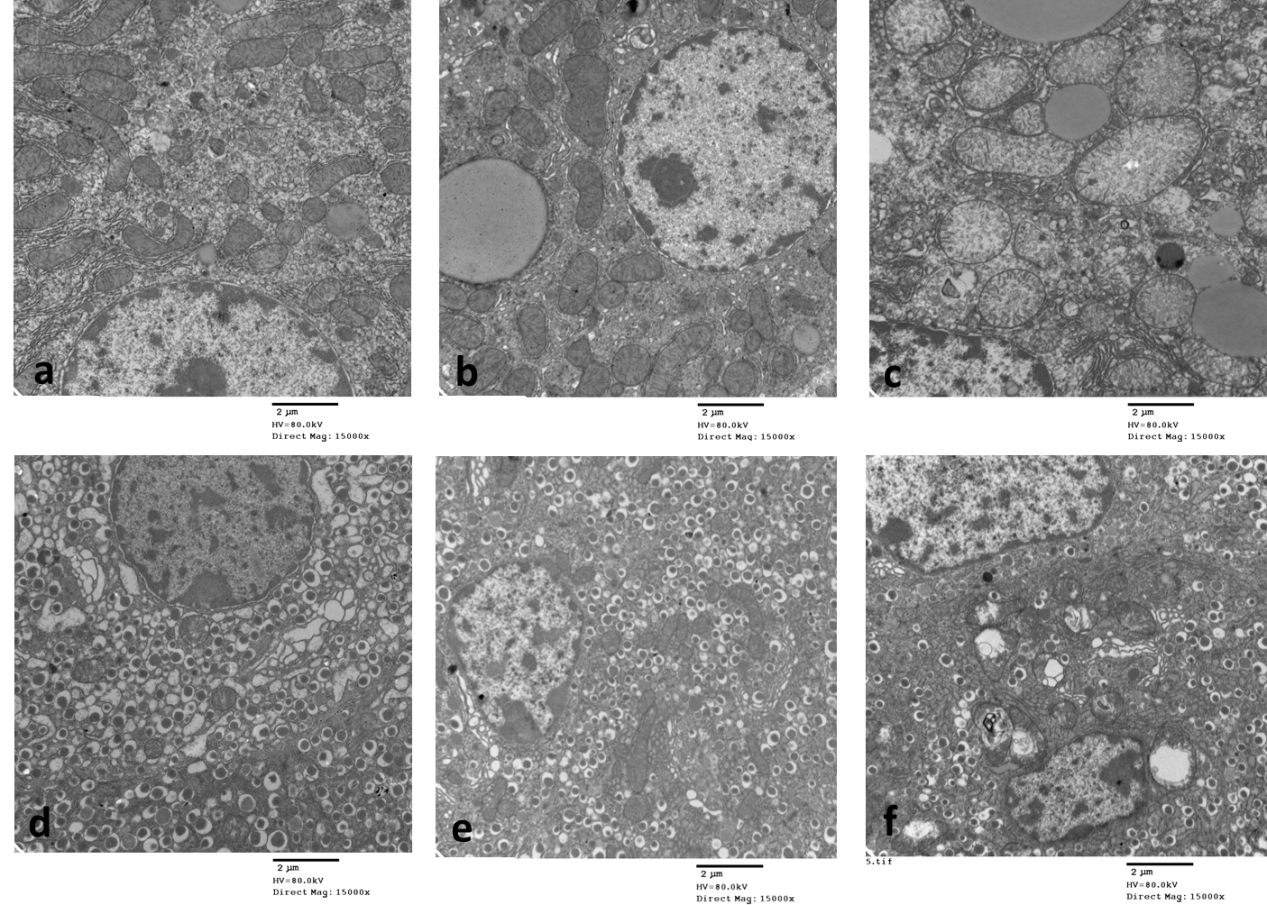


Fig. S4

Sig GO terms of DE gene-CC

Sig GO terms of DE gene-CC


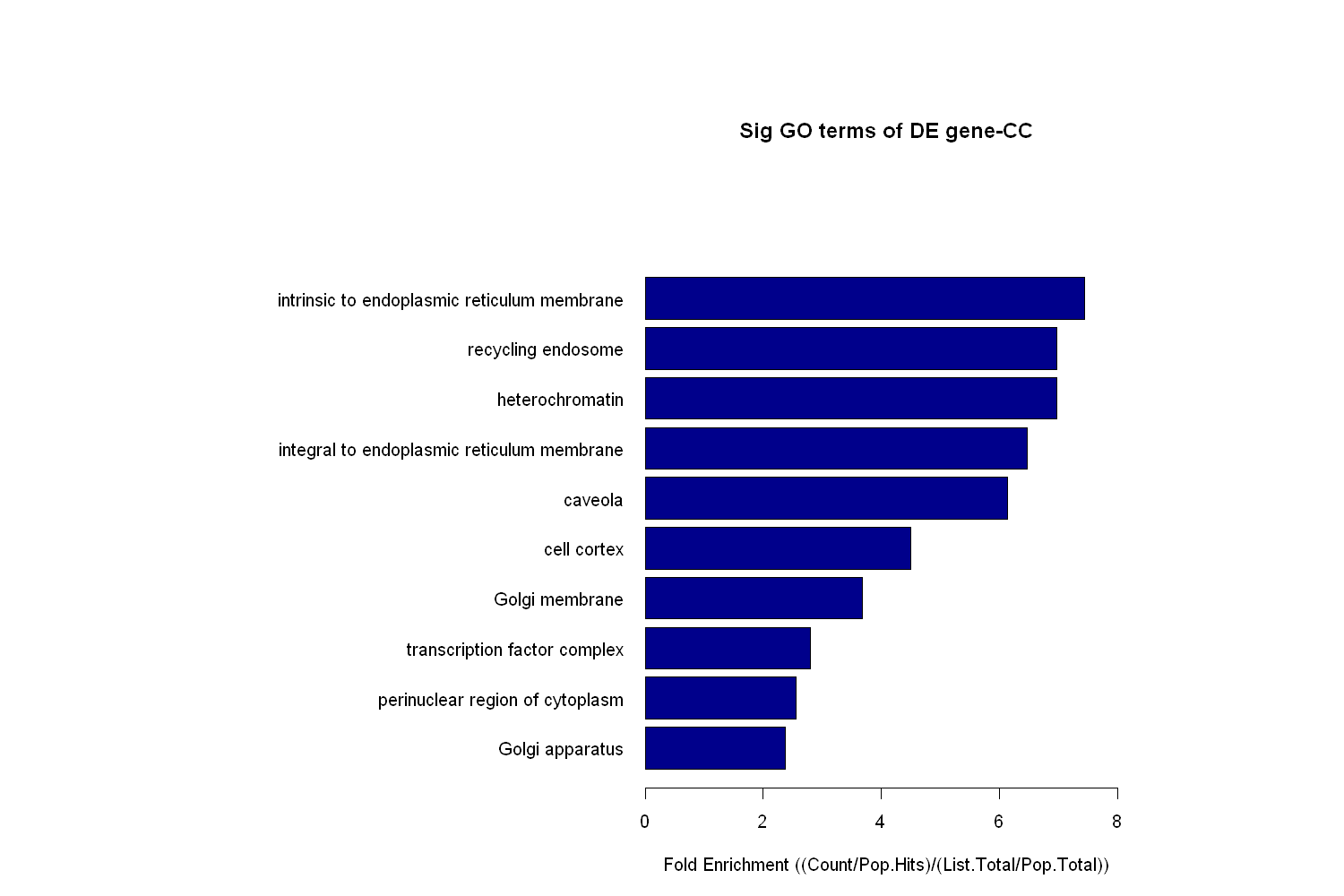


**a**


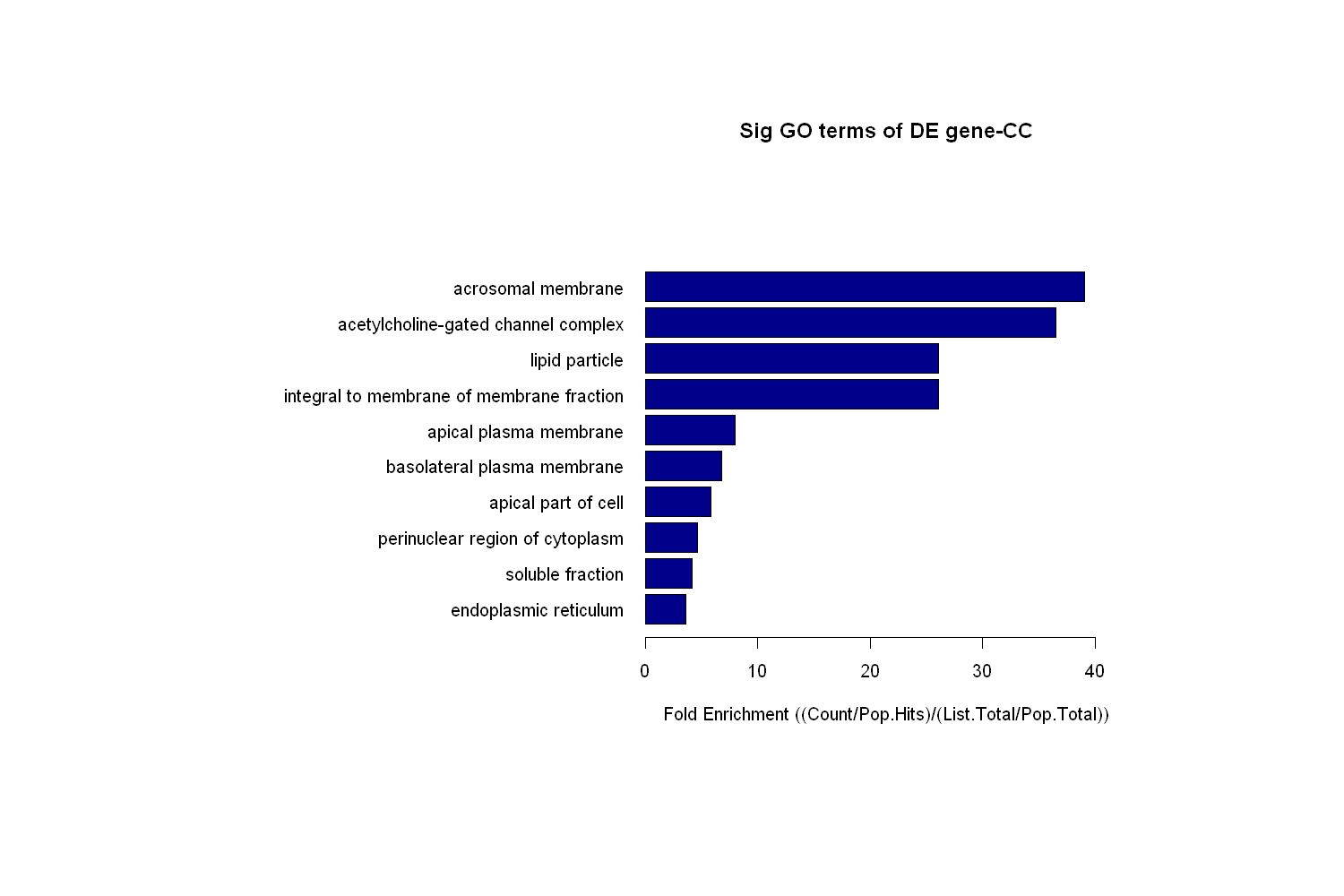

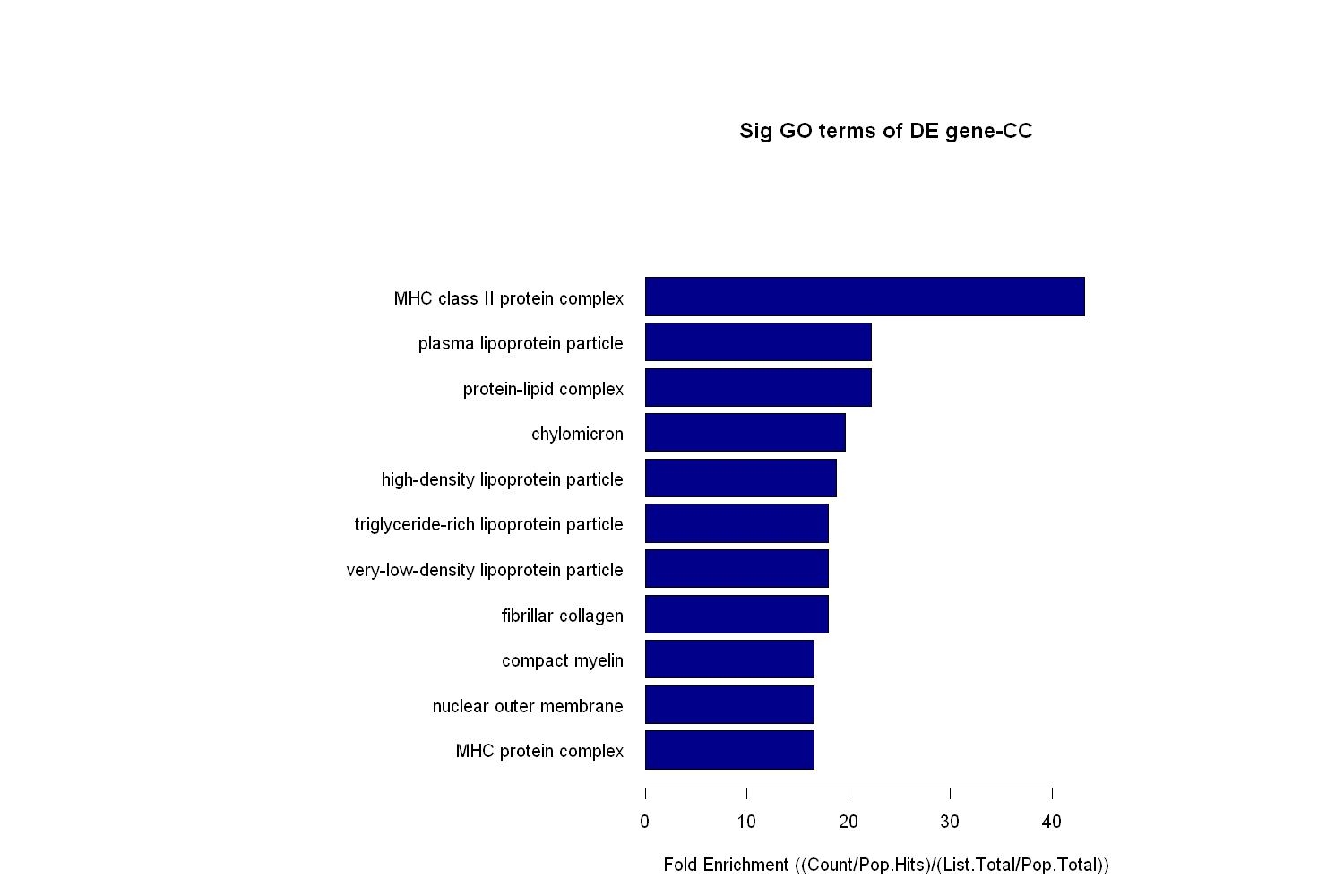

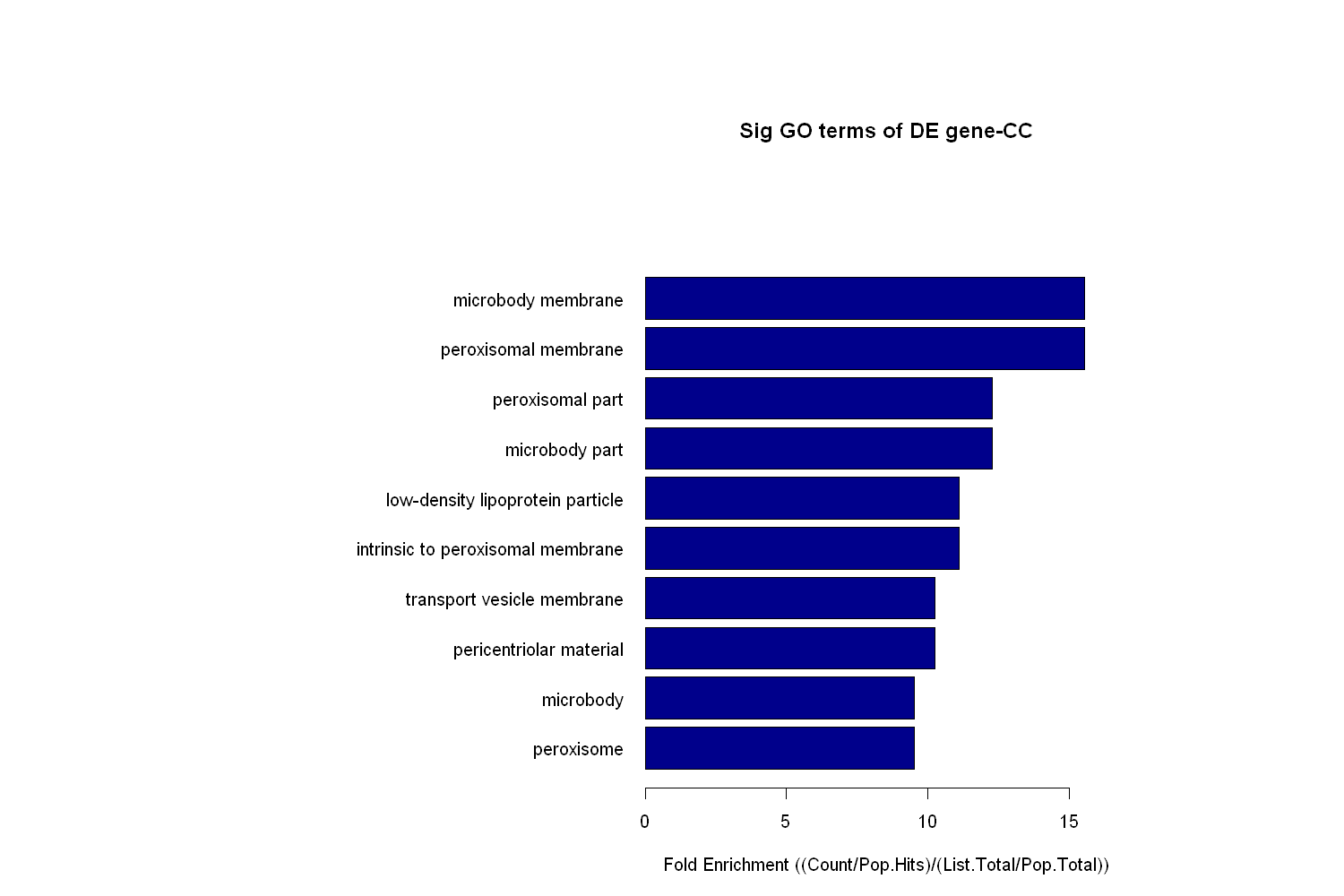

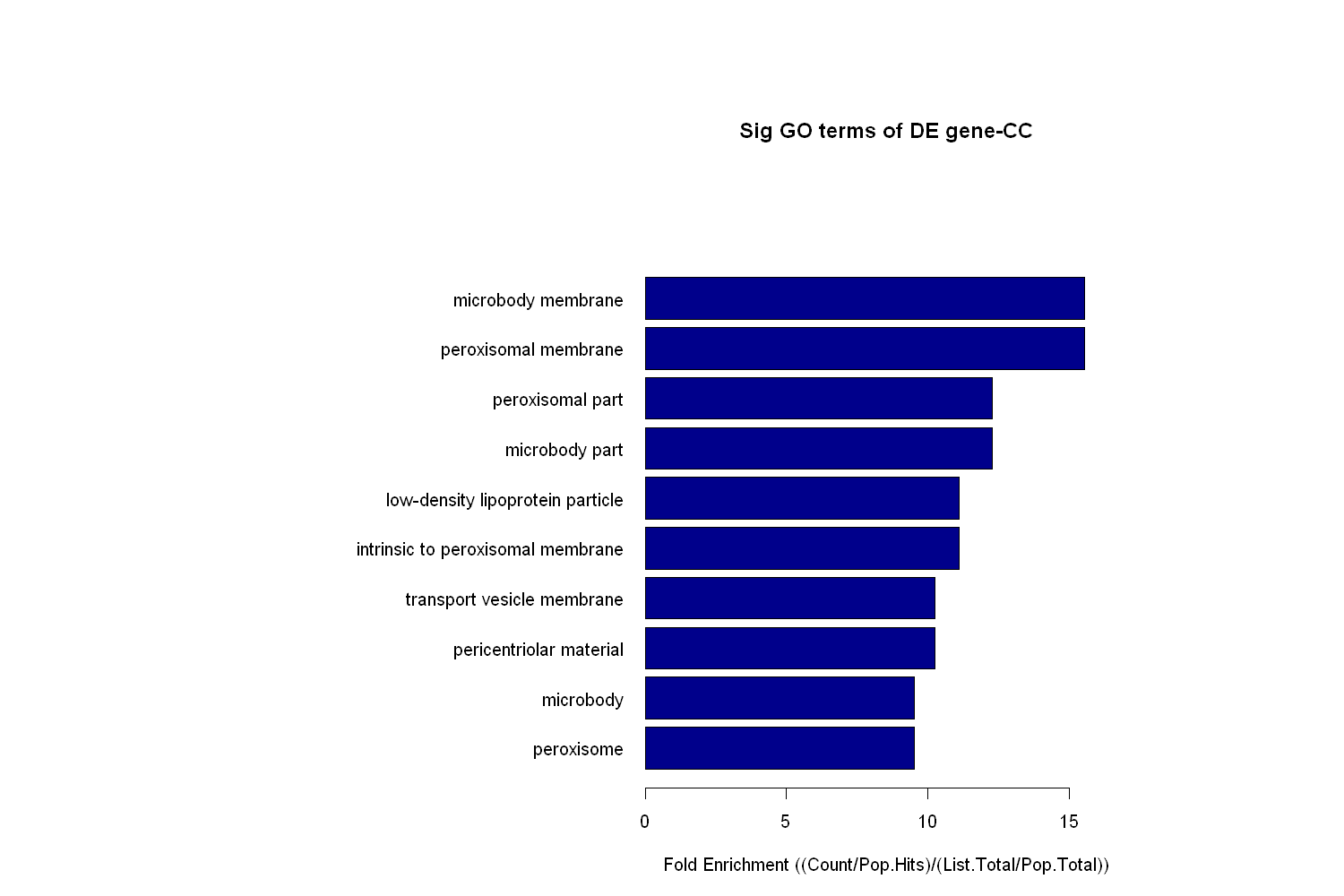

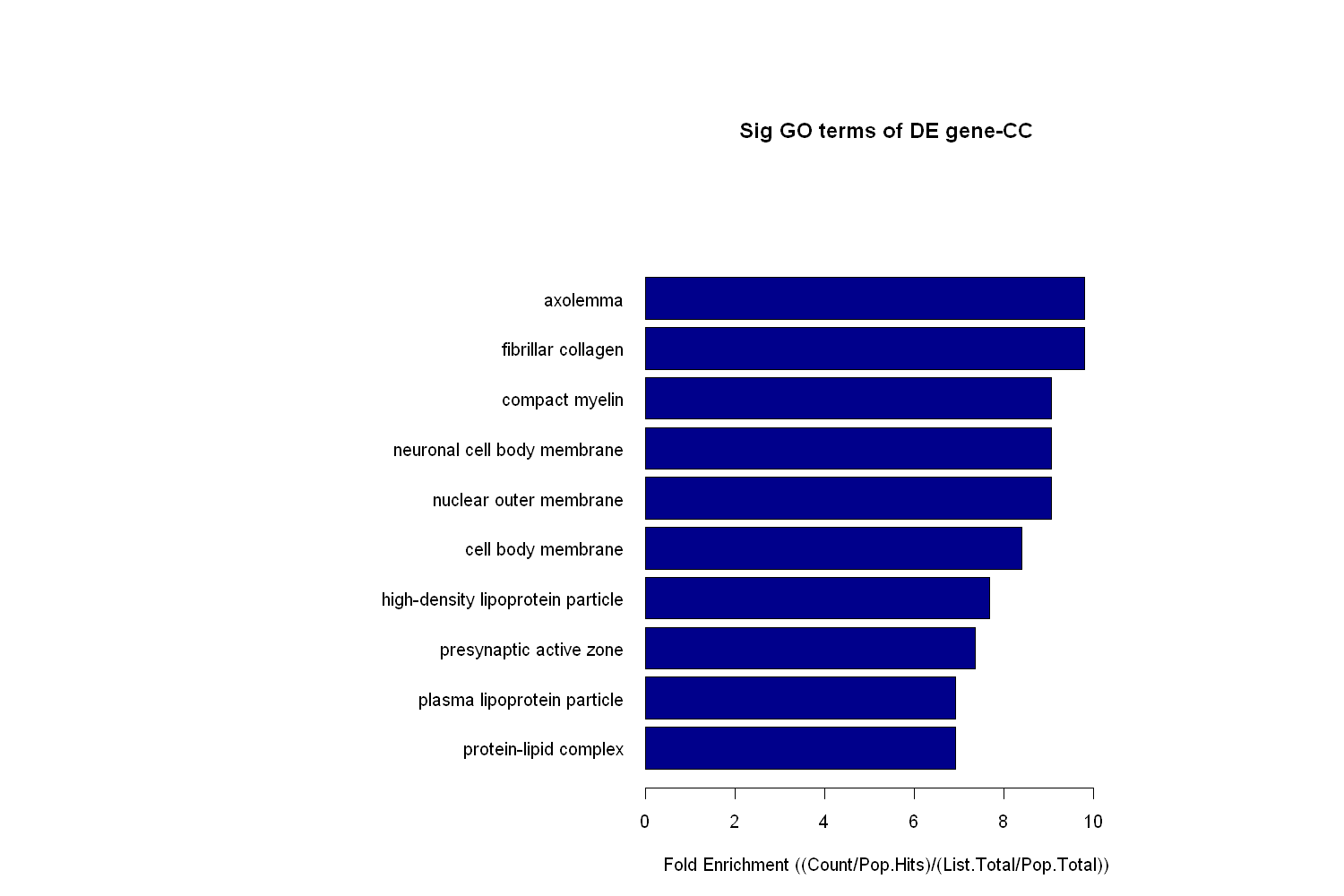

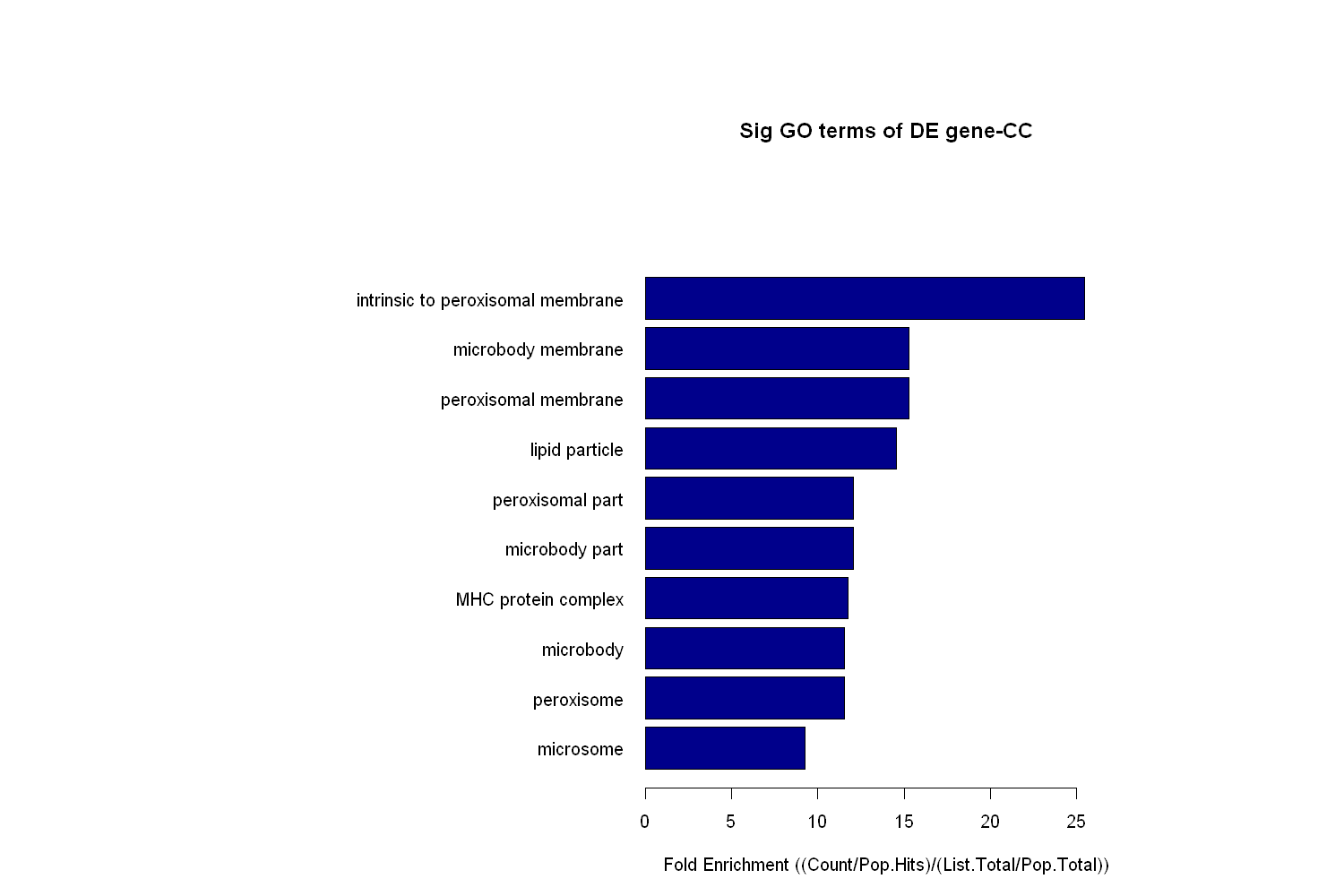

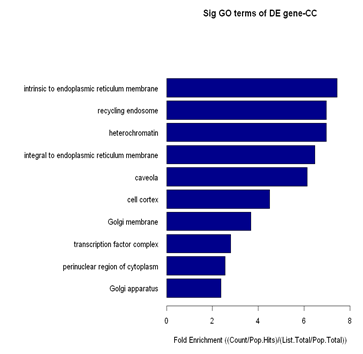

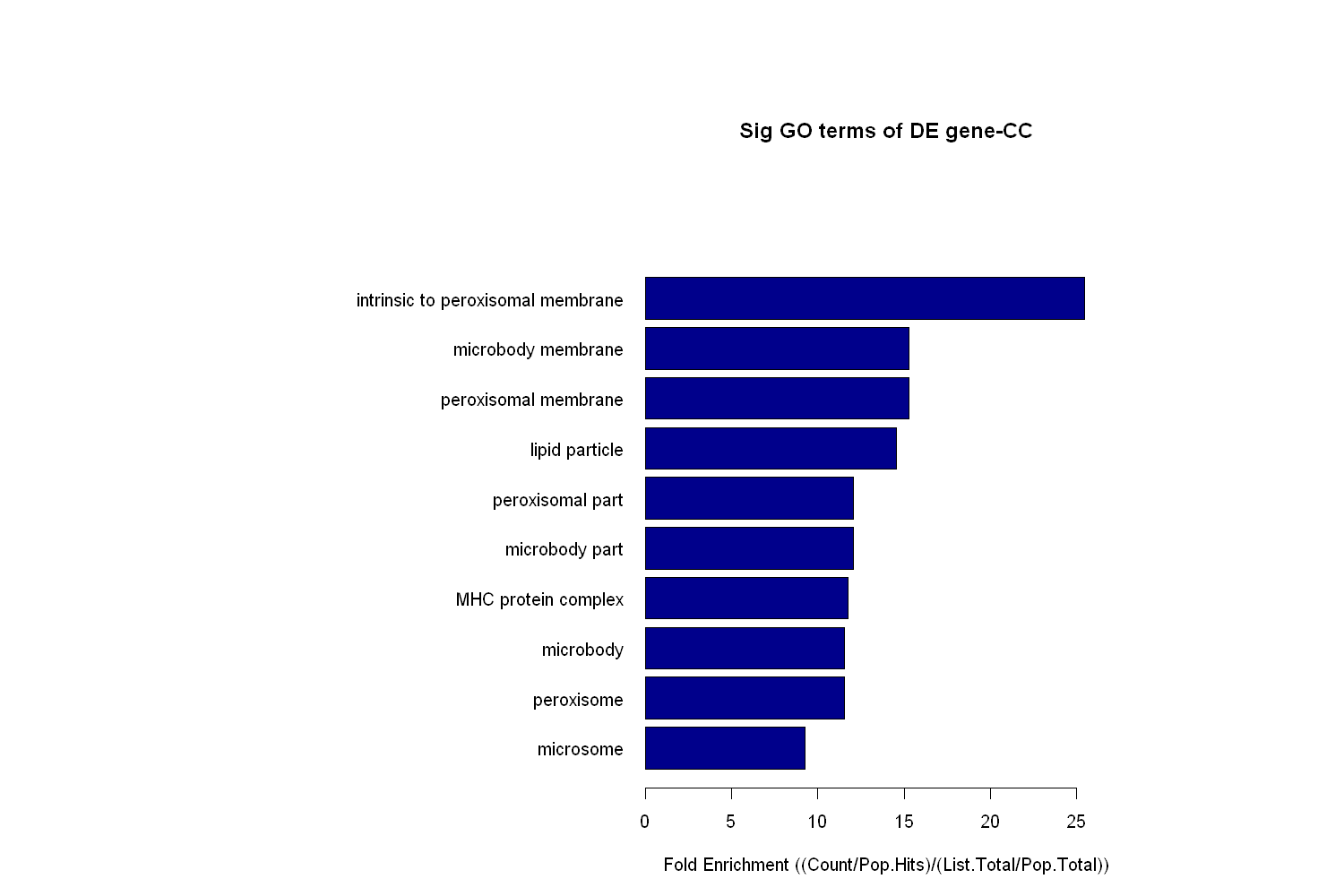

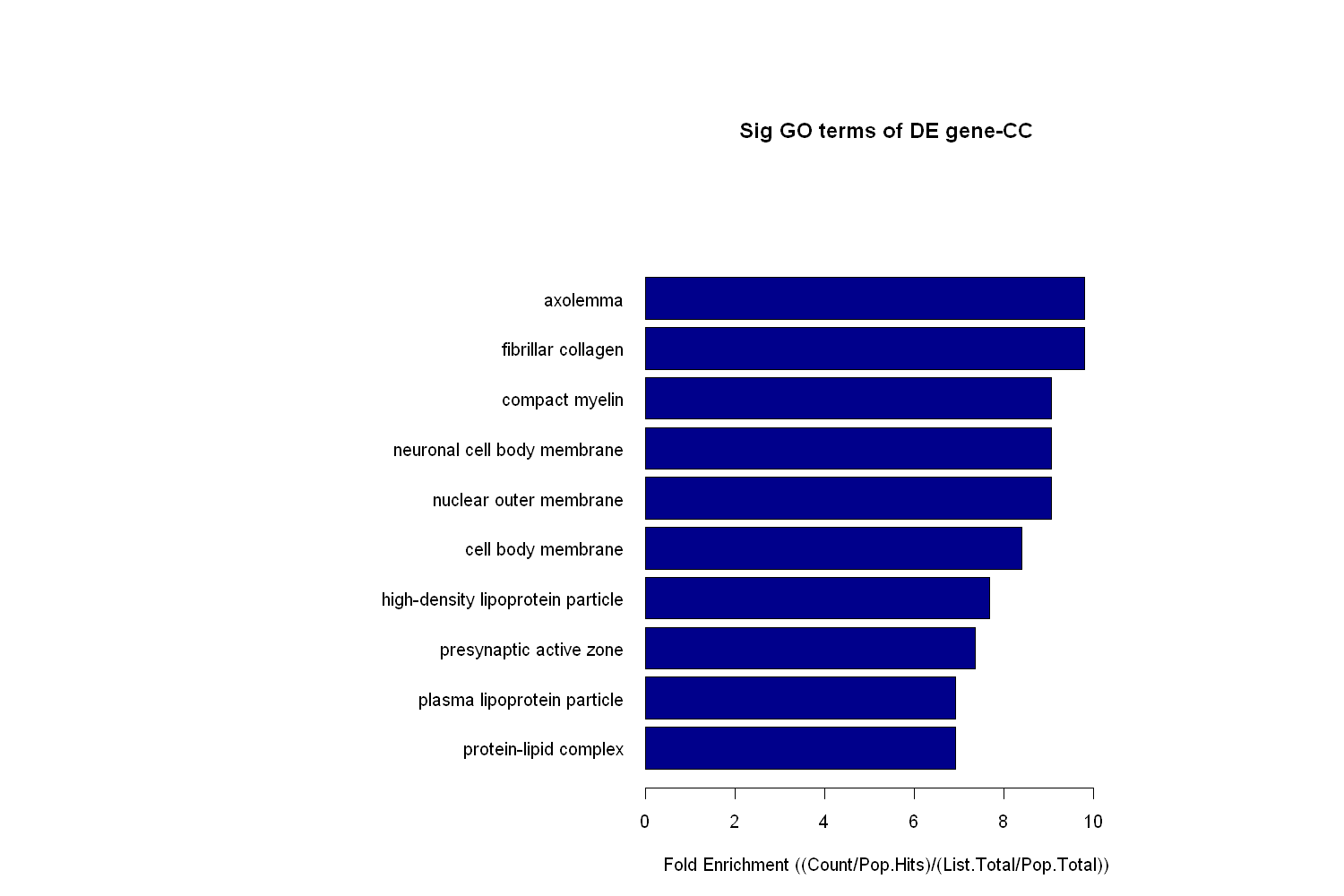

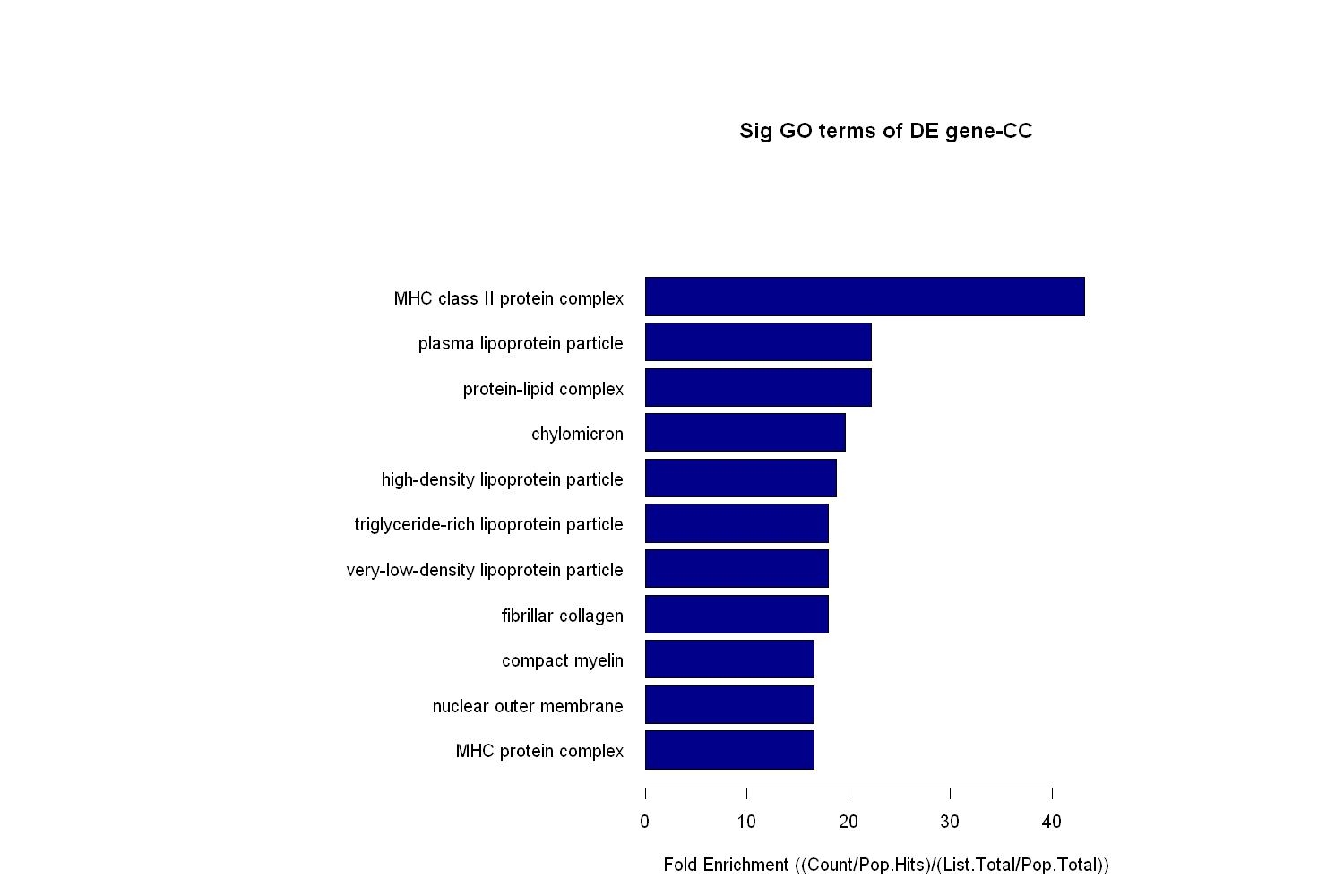

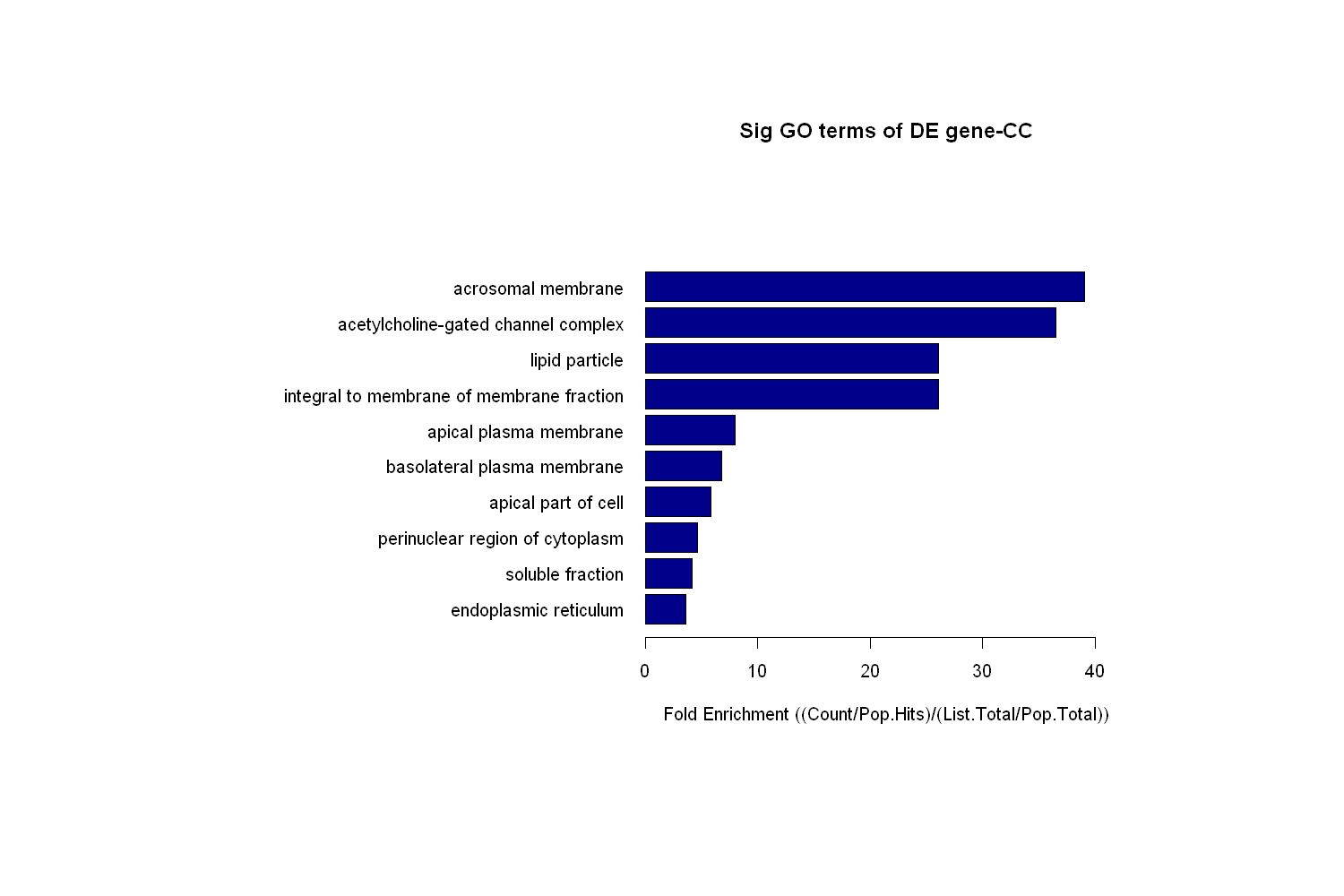


**b**

**c**

**d**

**e**

**f**

Fold Enrichment ((Count/Pop.Hits)/(List.Total/Pop.Total))

0 10 20 30 40

Fold Enrichment ((Count/Pop.Hits)/(List.Total/Pop.Total))

0 10 20 30 40

Sig GO terms of DE gene-CC

Sig GO terms of DE gene-CC

Fold Enrichment ((Count/Pop.Hits)/(List.Total/Pop.Total))

0 10 20 30 40

Fold Enrichment ((Count/Pop.Hits)/(List.Total/Pop.Total))

0 10 20 30 40

Fold Enrichment ((Count/Pop.Hits)/(List.Total/Pop.Total))

0 10 20 30 40

Fold Enrichment ((Count/Pop.Hits)/(List.Total/Pop.Total))

0 10 20

Sig GO terms of DE gene-CC

Sig GO terms of DE gene-CC


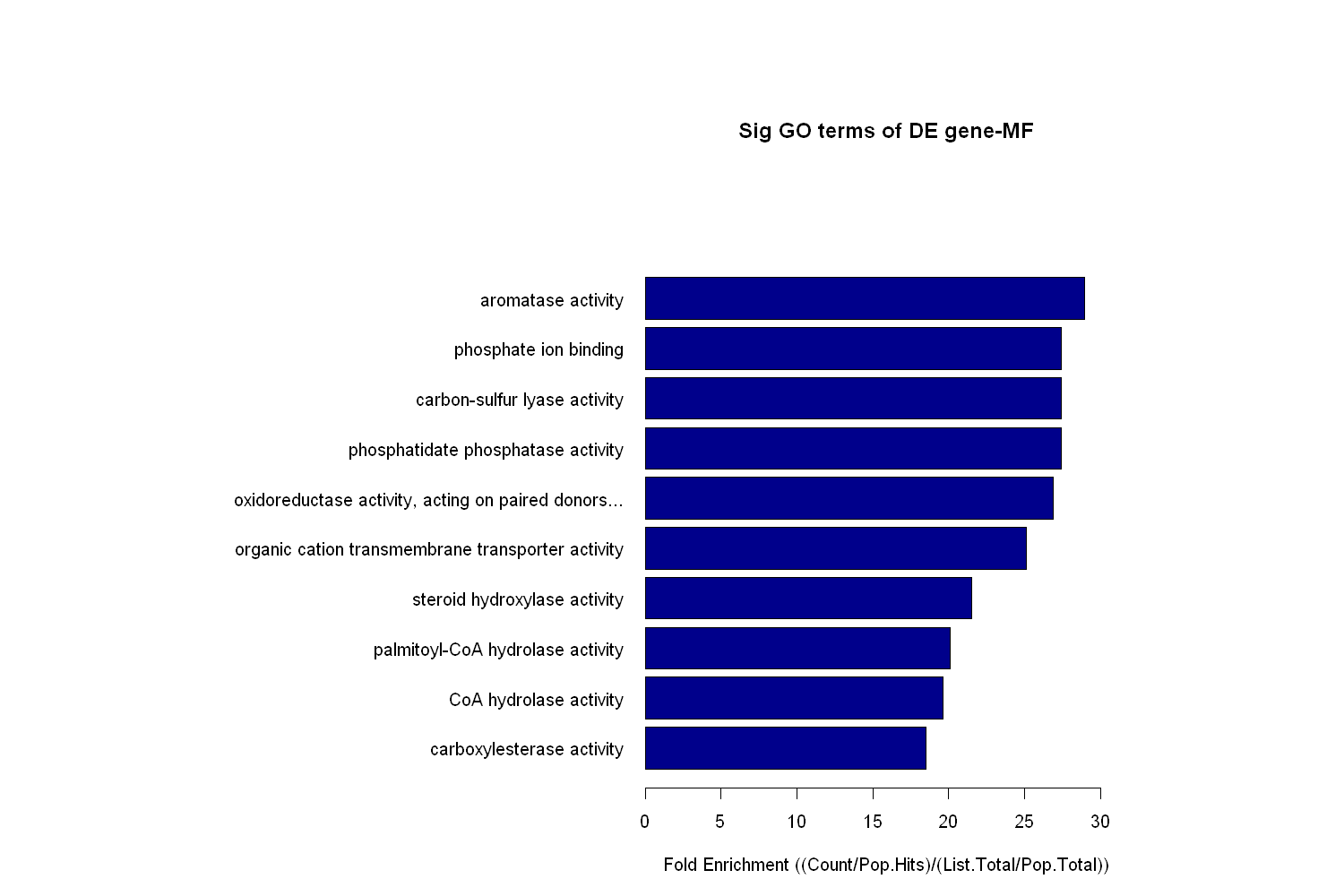
 Fig. S5

**a**

**b**

Sig GO terms of DE gene-MF


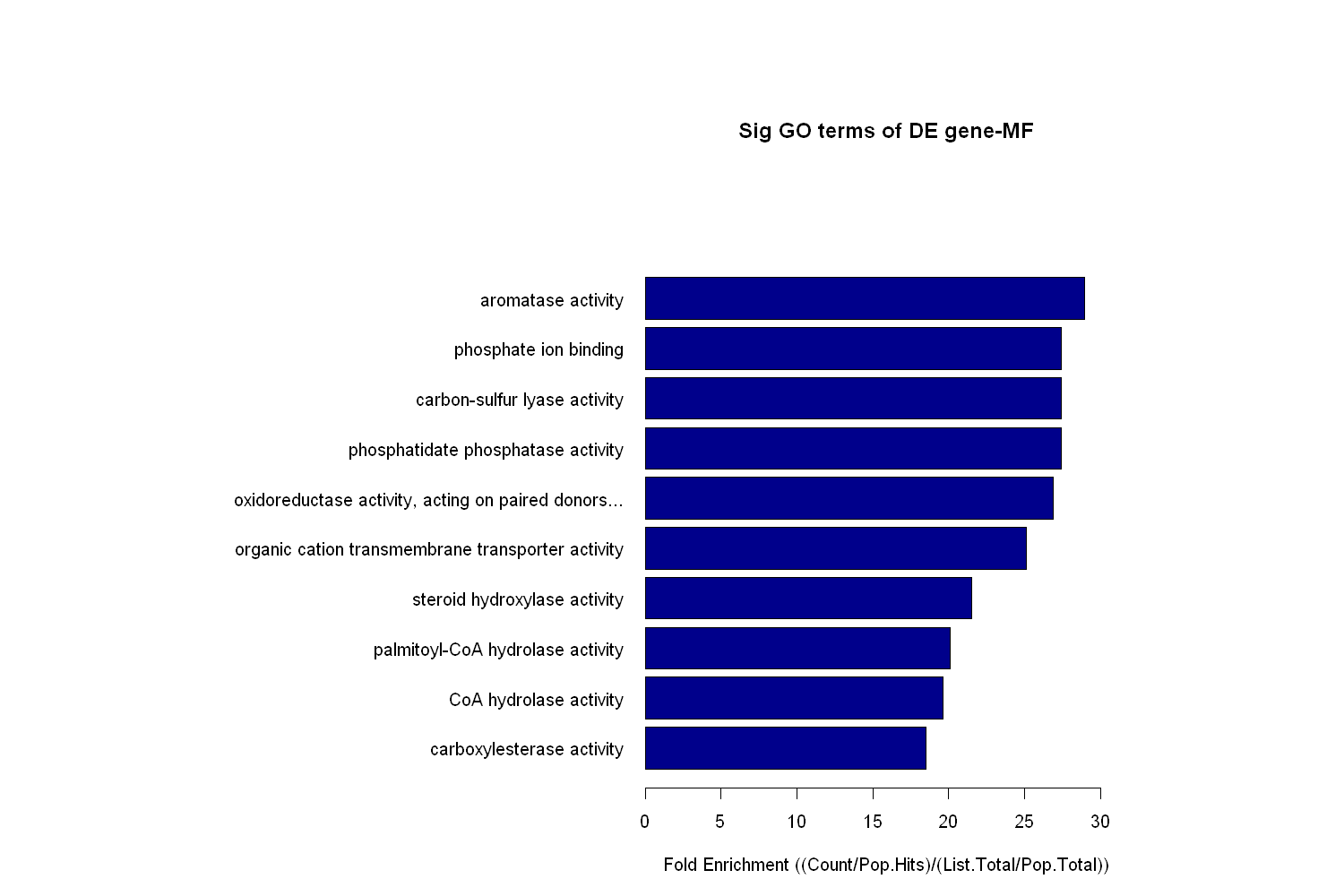

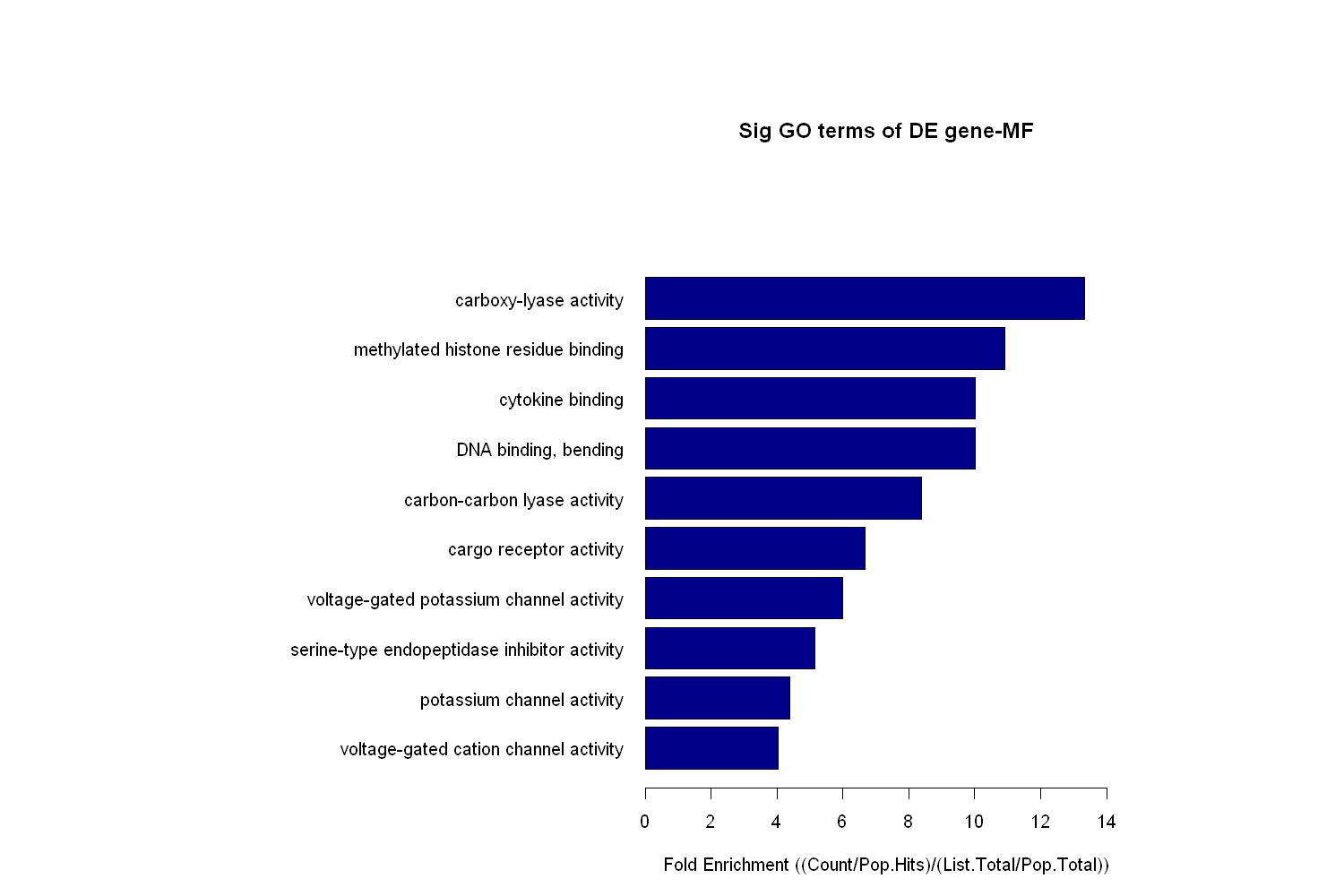

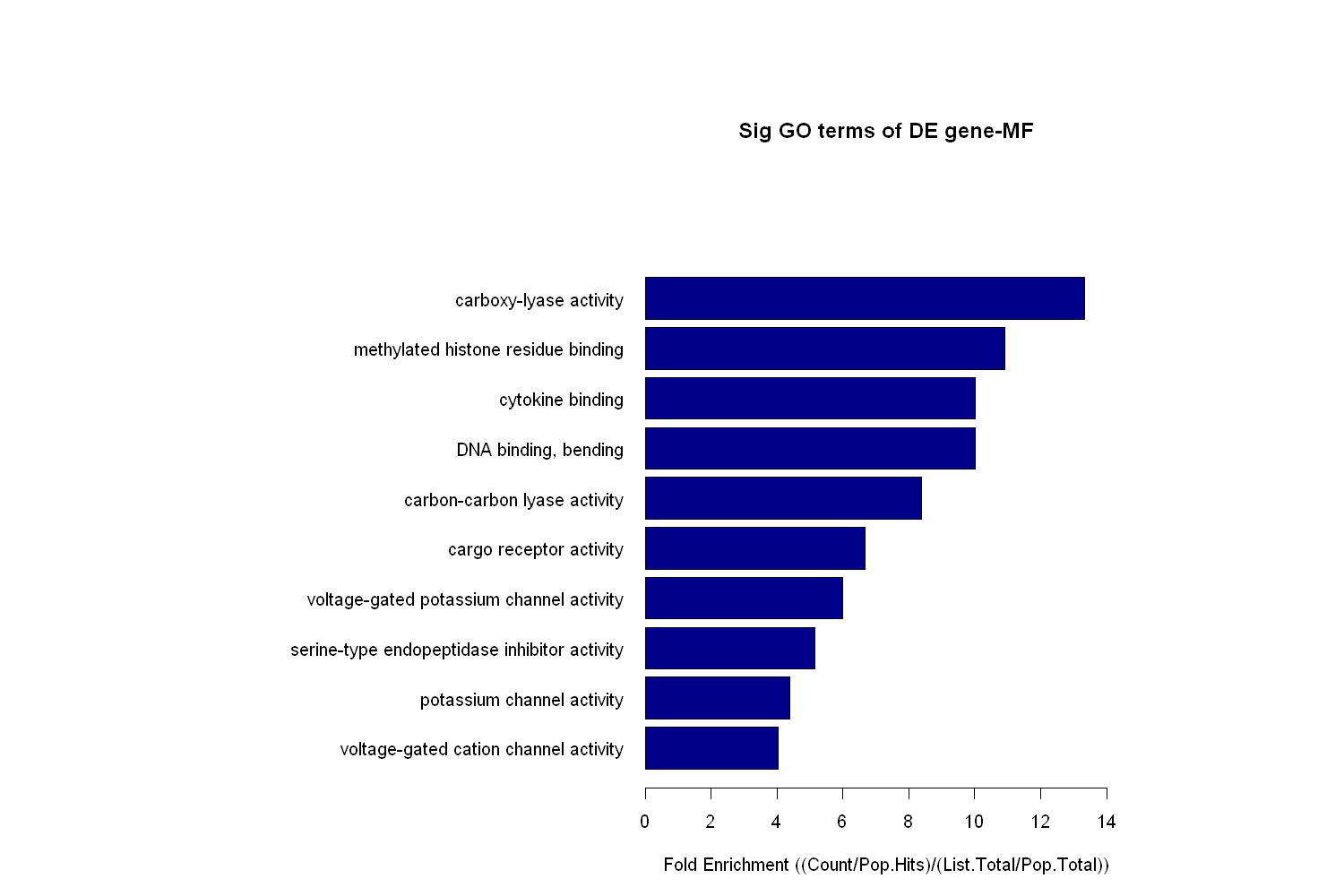


Sig GO terms of DE gene-MF


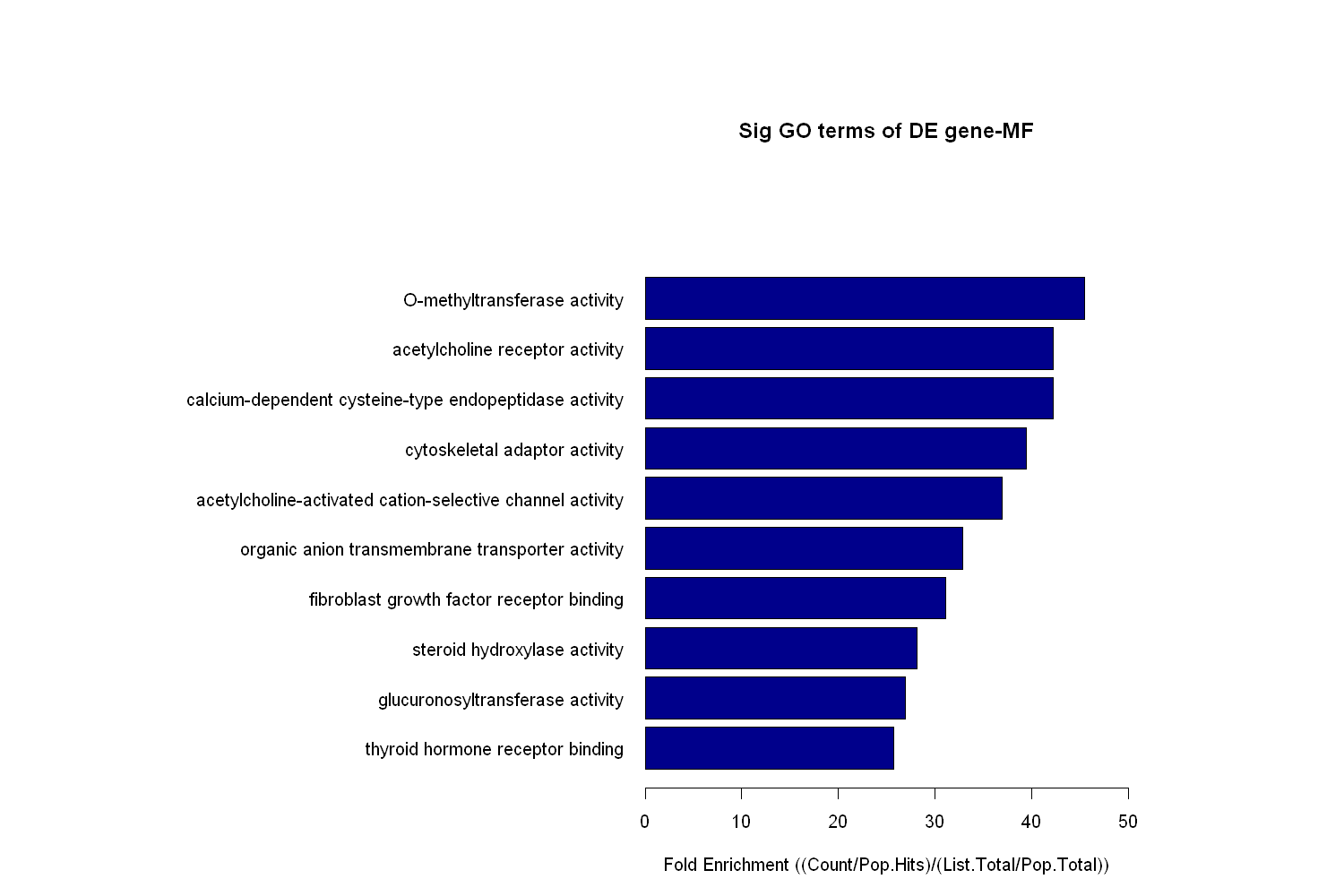

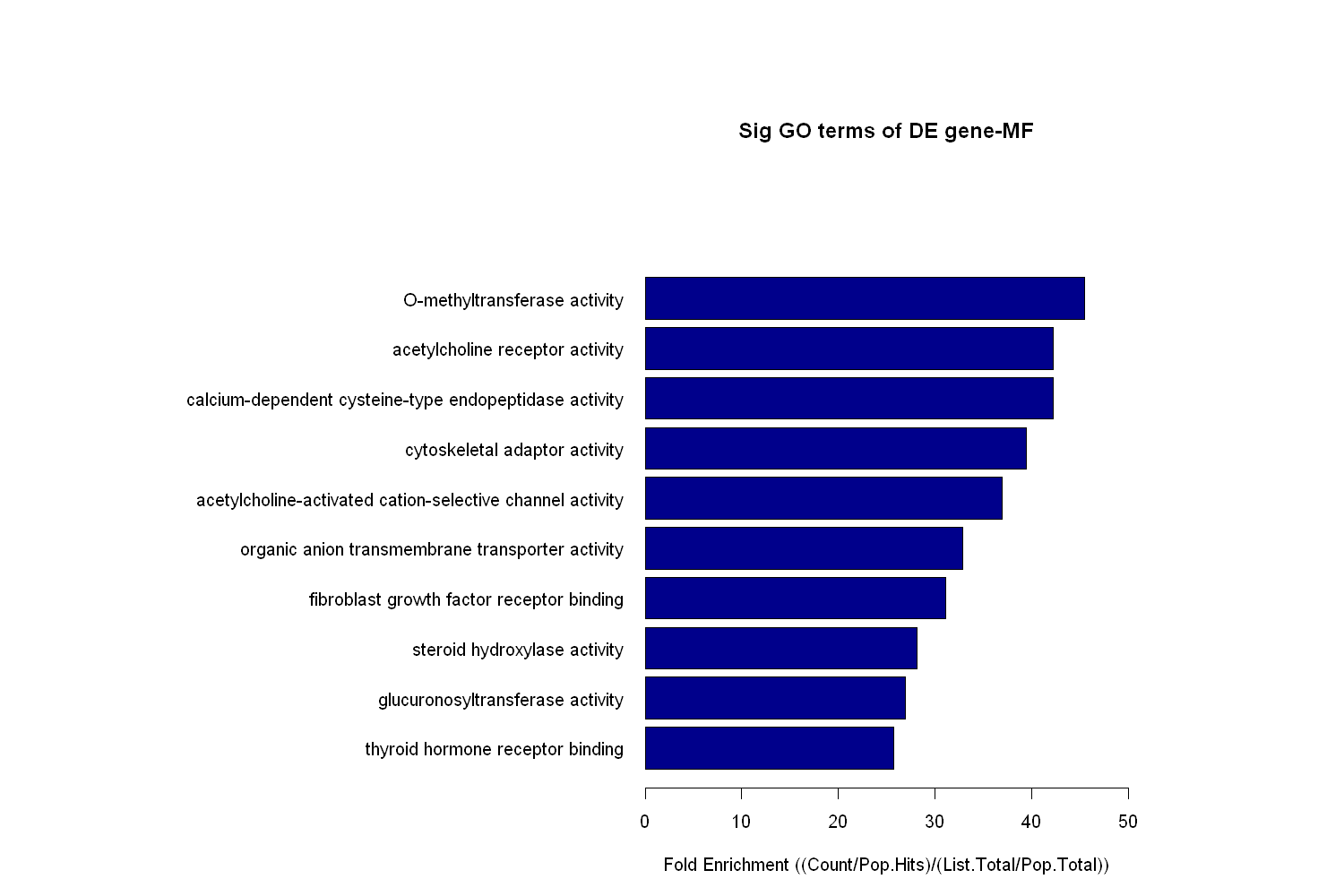

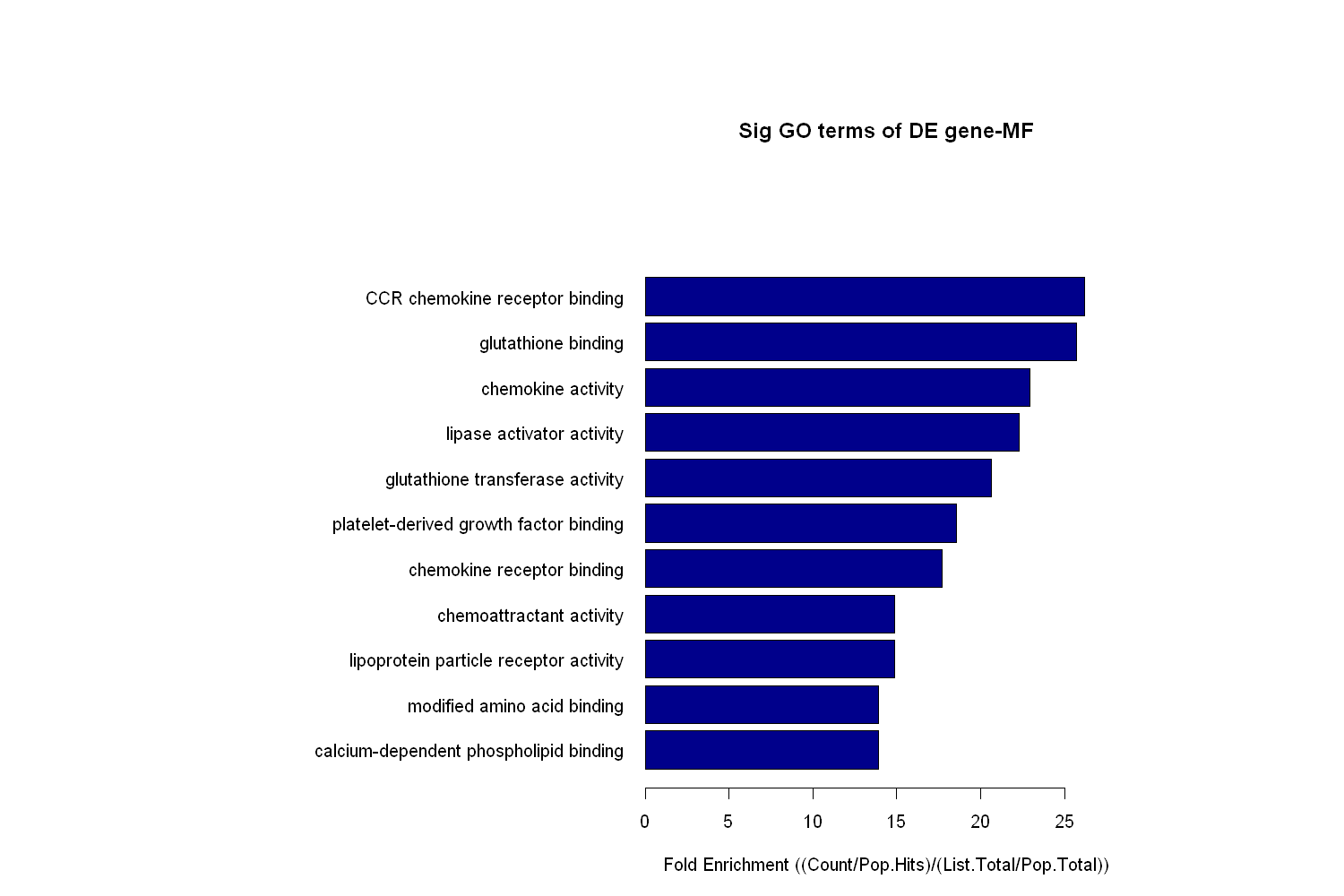

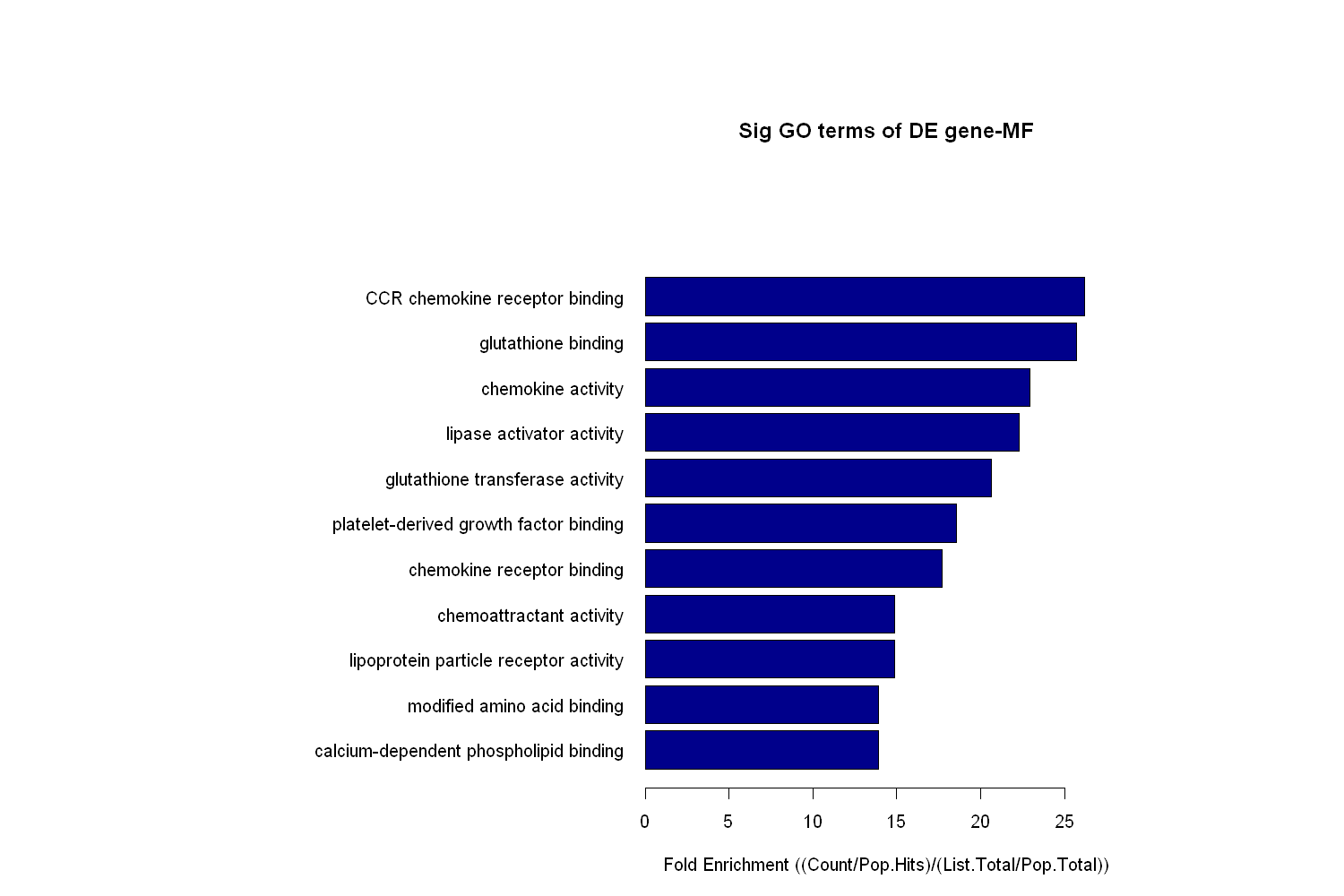

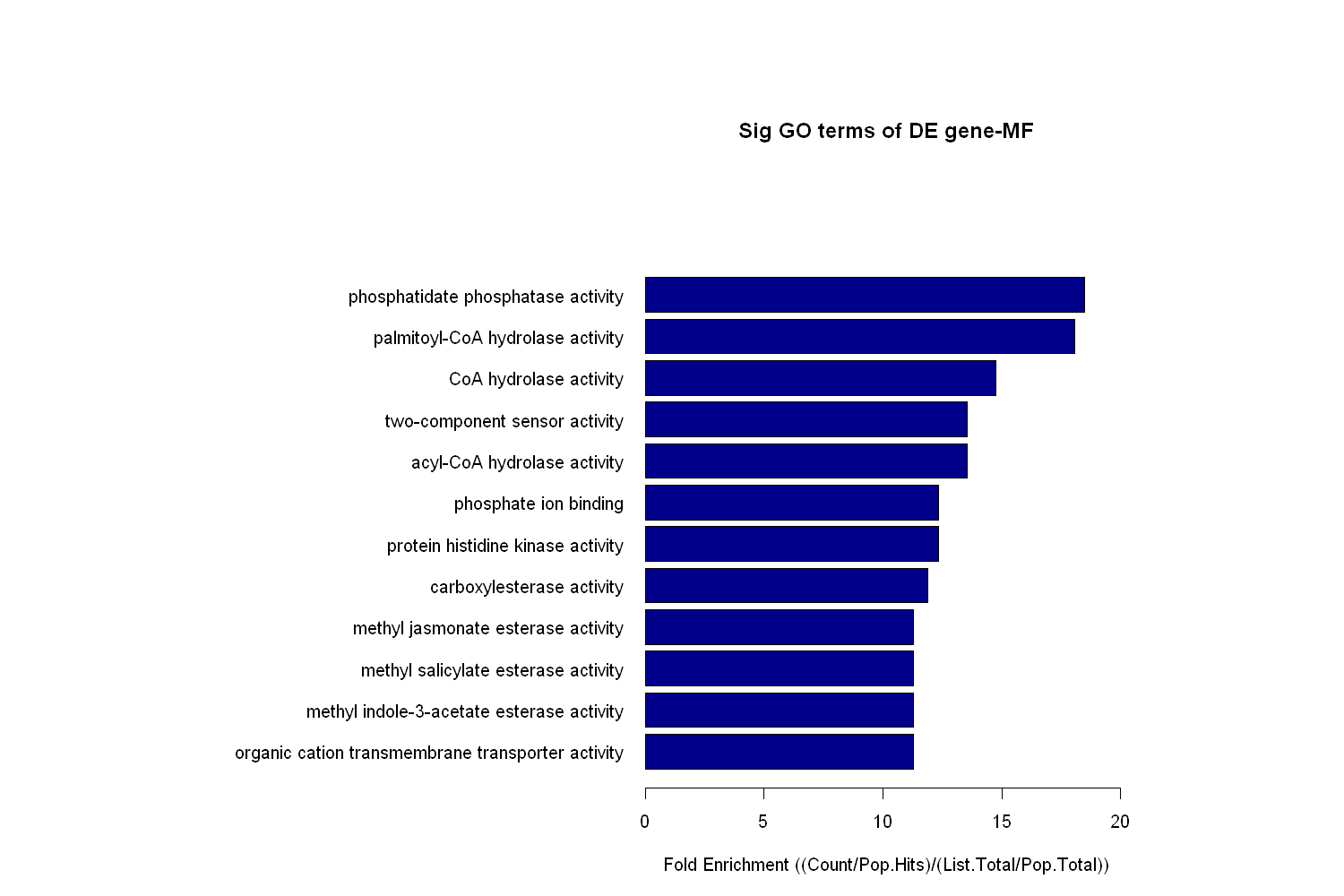

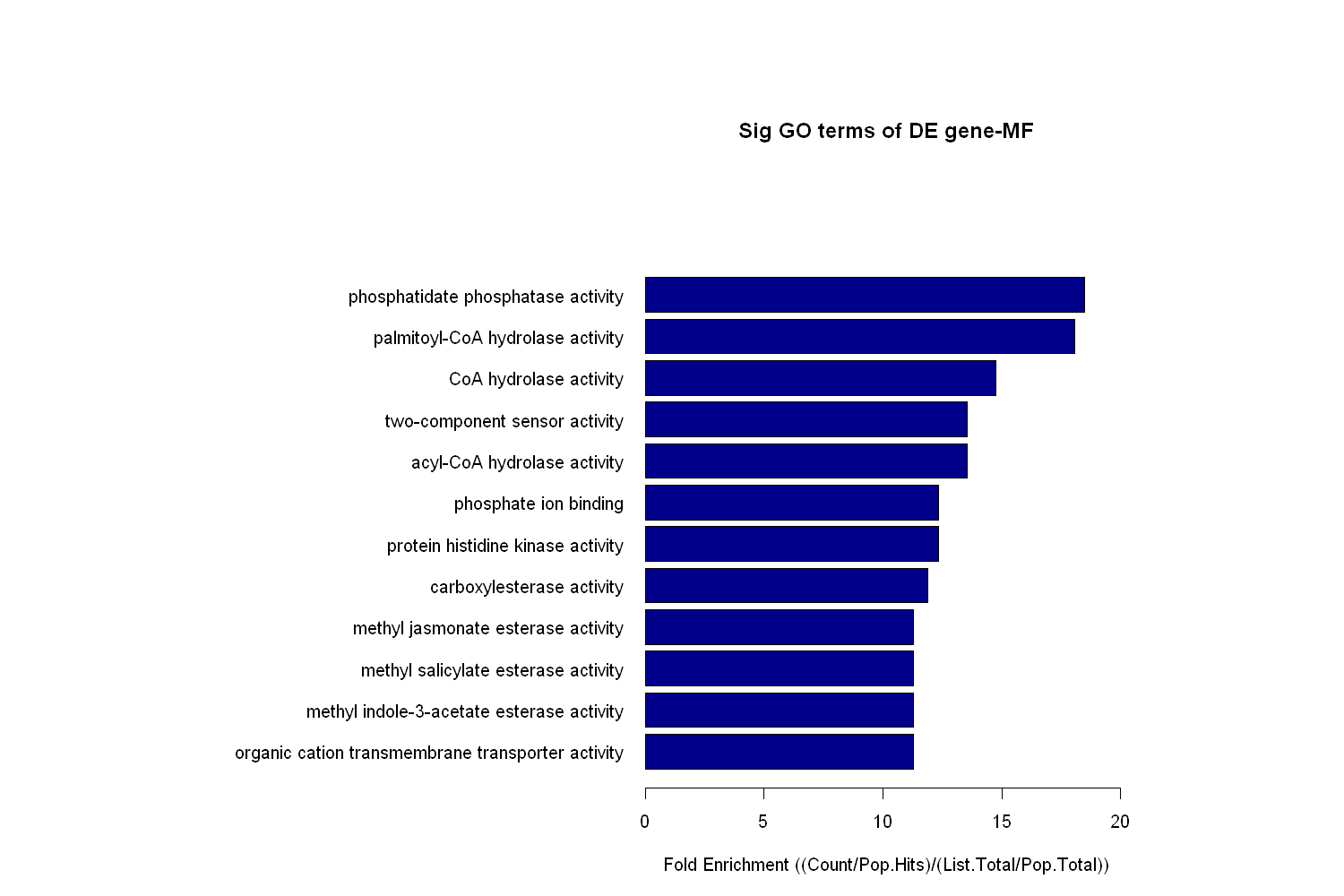

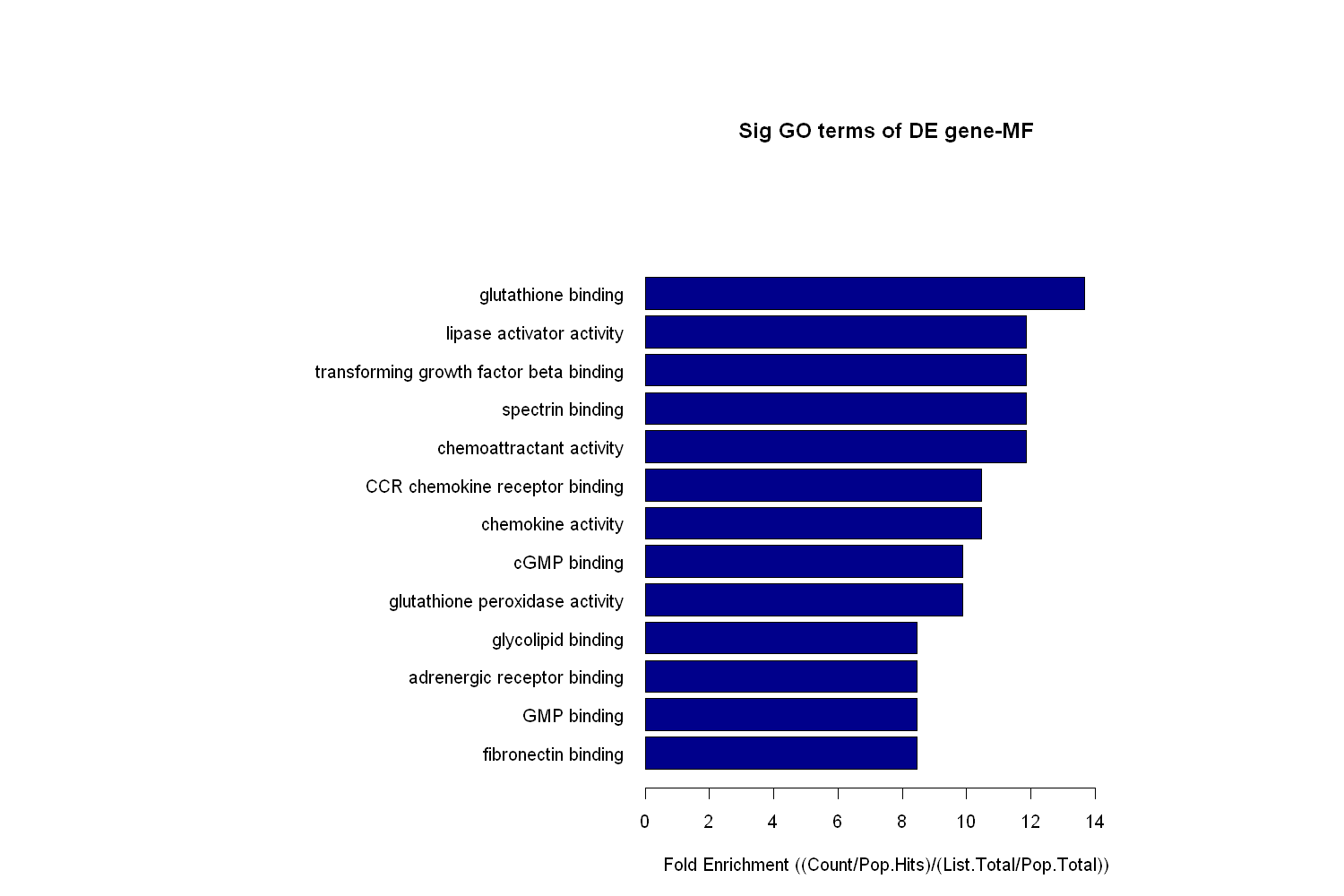

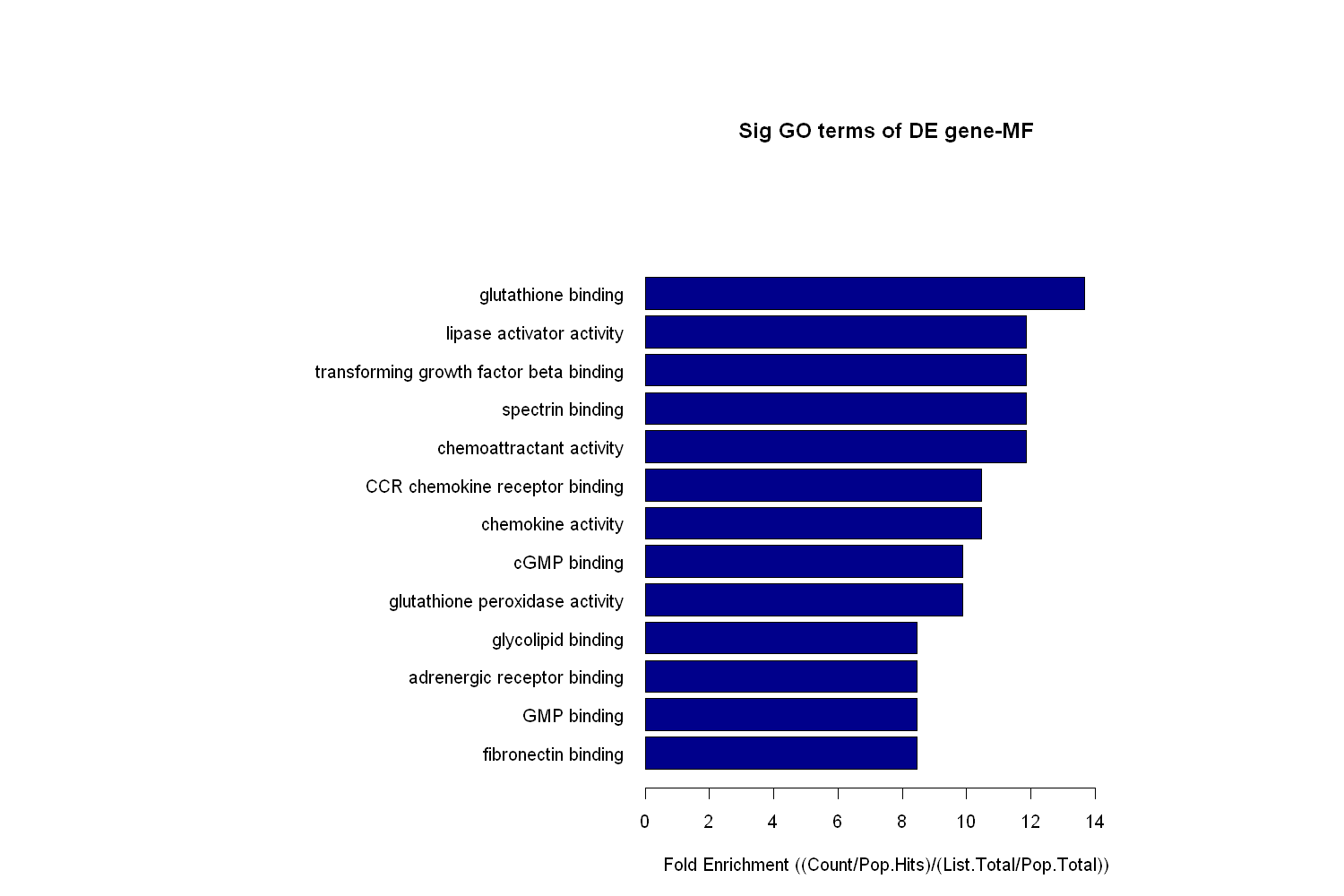


0 10 20 30 40

Fold Enrichment ((Count/Pop.Hits)/(List.Total/Pop.Total))

Sig GO terms of DE gene-MF

**f**

Sig GO terms of DE gene-MF

0 10 20 30 40

Fold Enrichment ((Count/Pop.Hits)/(List.Total/Pop.Total))

**e**

**c**

**d**

Fold Enrichment ((Count/Pop.Hits)/(List.Total/Pop.Total))

Sig GO terms of DE gene-MF

0 10 20 30 40

0 10 20 30 40

Fold Enrichment ((Count/Pop.Hits)/(List.Total/Pop.Total))

Sig GO terms of DE gene-MF

Fold Enrichment ((Count/Pop.Hits)/(List.Total/Pop.Total))

0 10 20 30 40

Fold Enrichment ((Count/Pop.Hits)/(List.Total/Pop.Total))

0 10 20 30 40
